# UAP1 as a prognostic biomarker regulating malignant biological functions in multiple myeloma cells

**DOI:** 10.3389/fonc.2026.1902414

**Published:** 2026-07-22

**Authors:** Xiang Li, Changjian Yan, Lihua Lu, Xiaoyu Wei, Qing Cai, Yanxin Chen, Jianda Hu

**Affiliations:** 1 The Second Affiliated Hospital of Fujian Medical University, Quanzhou, China; 2 Institute of Precision Medicine, Fujian Medical University, Fuzhou, China; 3 Department of Gastroenterology and Hepatology, Bishan Hospital of Chongqing Medical University, Chongqing, China; 4 Fujian Medical University Union Hospital, Fuzhou, China; 5 Department of Hematology, Zhangpu County Hospital, Zhangzhou, China

**Keywords:** biomarker, multiple myeloma, prognosis, treatment, UAP1

## Abstract

**Background:**

Multiple myeloma (MM) is a hematological malignancy characterized by proliferation of malignant plasma cells. UDP-N-acetylglucosamine pyrophosphorylase 1 (UAP1) has been shown to promote progression in various cancers. However, its specific role in MM remains unclear.

**Methods:**

We analyzed six datasets comprising 3,117 patients from the Multiple Myeloma Research Foundation and Gene Expression Omnibus databases. The reliability of UAP1 as a prognostic predictor was also systematically evaluated. To further elucidate the specific mechanisms underlying the role of UAP1 in promoting MM progression, we performed an in-depth analysis of single-cell RNA sequencing data from 24 patients with MM. Additionally, we evaluated UAP1’s therapeutic potential using two MM cell lines (RPMI-8226 and U266).

**Results:**

Across all 3,117 patients with MM, high UAP1 expression correlated with significantly worse overall survival, event-free survival, and progression-free survival. Patients with high UAP1 expression consistently exhibited concurrent genetic abnormalities. Single-cell data suggest that UAP1 is predominantly expressed in tumor cells, is closely associated with tumor cell differentiation signatures, and shows a correlative link with the expression of immunosuppressive pathway components. *In vitro* experiments demonstrated that UAP1 knockdown significantly inhibited cell proliferation and cell cycle progression, enhanced apoptosis, and increased the dexamethasone sensitivity of RPMI-8226 and U266 cells. Genetic knockdown of UAP1 significantly increased the proportion of CD8^+^ T cells in *in vitro* co-culture system with MM cells.

**Conclusions:**

UAP1 expression is significantly correlated with tumor differentiation, proliferation, immunosuppression, and patient prognosis, establishing it as an prognostic biomarker Regulating Malignant Biological Functions in Multiple Myeloma Cells. Our findings may guide the development of novel treatment strategies and improve clinical management.

## 1 Introduction

Multiple myeloma (MM) is a plasma cell disorder characterized by clonal proliferation in the bone marrow, leading to excess monoclonal immunoglobulins and cytokine-mediated skeletal damage. Its clinical features include bone lesions, hypercalcemia, anemia, and renal impairment (1). Prognostic markers have evolved from hemoglobin, calcium, and creatinine to include β2-microglobulin (β2M), albumin, C-reactive protein, and plasma cell proliferation index (2).

Although the molecular understanding of MM has advanced, it remains highly heterogeneous (3). Sequencing studies have identified common mutations in KRAS, NRAS (~20% each), TP53, DIS3, FAM46C, and BRAF (~10% each); however, clonal diversity limits prognostic biomarker utility (4). Metabolic reprogramming including glycosylation promotes tumorigenesis, metastasis, and therapy resistance (5–9). The hexosamine biosynthesis pathway (HBP) is linked to tumorigenesis (10), and features the overexpression of UDP-N-acetylglucosamine pyrophosphorylase 1 (UAP1), which elevates HBP end products and enhances cancer invasion and metastasis (11–15).

Previous studies have demonstrated that UAP1 is associated with solid tumors such as bladder, prostate, and lung cancer and osteosarcoma (13, 14, 16, 17), mechanistically through its regulation of the HBP. For example, UAP1 is overexpressed in prostate cancer, aids in stress resistance and progression (18), and is correlated with advanced lung adenocarcinoma (14). Its knockdown suppresses the proliferation, invasion, and migration of bladder cancer (13). However, the role of UAP1 in hematological malignancies remains unexplored, and few studies have investigated its relevance in MM.

Therefore, we analyzed data from 3,117 MM patients from the Gene Expression Omnibus (GEO) and the Multiple Myeloma Research Foundation (MMRF) to evaluate UAP1 expression as an independent prognostic predictor. We also analyzed single-cell samples from 24 patients with MM (19) (Data from Liu et al.) to further evaluate the function of UAP1. Additionally, we explored the potential of UAP1 as a therapeutic target for MM through *in vitro* experiments using siRNA technology. This study may provide new insights into MM progression mechanisms and offer novel strategies for prognosis and treatment.

## 2 Materials and methods

### 2.1 Data source and analysis

We analyzed RNA expression profiles from 3,117 MM patients from two databases: 843 from MMRF and 2,274 from GEO (including GSE2658, GSE24080, GSE9782, GSE136337, GSE57317, and GSE4581, all using CD138^+^ bone marrow plasma cells). Using these integrated datasets, we evaluated the correlation between UAP1 expression and overall survival. All original studies were approved by the local ethics committee and all participants signed informed consent forms. Our study was conducted following relevant guidelines and regulations.

### 2.2 Gene expression analysis

All GEO datasets were downloaded as raw/series matrix files directly from public databases and processed independently according to the standard pipelines specific to each platform. For microarray expression data, the Robust Multi-array Average (RMA) method was applied for background correction and quantile normalization, followed by log2 transformation. RNA-seq data from the MMRF cohort were presented as RPKM values and incorporated into the analysis after log2(RPKM + 1) transformation. All samples were CD138^+^ bone marrow plasma cells, ensuring consistent sample type across the datasets. Within each cohort, the optimal cutoff for UAP1 expression was determined using the surv_cutpoint function, which stratified patients into high- and low-expression groups. Given that different datasets originated from distinct platforms (e.g., Affymetrix U133 series vs. RNA-seq), merging the raw expression matrices across platforms would introduce batch effects that are difficult to correct. Therefore, rather than combining all datasets into a single matrix, we performed independent preprocessing and survival analysis for each cohort, thereby fundamentally circumventing cross-dataset batch effects. Normalization was carried out separately within each dataset. The GSE24080 dataset served as the discovery cohort, while independent validation was performed using multiple datasets including GSE2658, GSE9782, GSE136337, GSE57317, GSE4581, and the MMRF cohort. The primary clinical endpoints evaluated were event-free survival (EFS), progression-free survival (PFS), and overall survival (OS).

### 2.3 Univariate and multivariate Cox regression analyses

Survival analyses were performed independently within each individual cohort that had detailed clinical information. For each cohort, univariate Cox proportional hazards models were first constructed for every clinical and molecular variable (including ISS stage, age, sex, B2M, CRP, LDH, ALB, HGB, ASPC, BMPC, MRI, cytogenetic abnormalities, and FISH 1q21 amplification) as well as for UAP1 expression groups. Hazard ratios (HRs), 95% confidence intervals (CIs), and P values were calculated for each variable. Variables with P < 0.05 in the univariate analysis were then entered into a multivariate Cox model to adjust for potential confounders and to determine whether UAP1 served as an independent prognostic factor. Continuous laboratory indicators were dichotomized according to their median values, while variables with missing data were included in their continuous form to avoid complete case exclusion. This analytical strategy was applied to GSE24080 as the discovery cohort, with the remaining cohorts used for validation purposes.

### 2.4 Meta-analysis across multiple cohorts with forest plot

To evaluate the reproducibility and robustness of the prognostic value of UAP1 across different datasets, we extracted the log(HR) and its standard error for UAP1 (high vs. low) from each independent cohort as effect sizes. These estimates were then pooled using a random-effects model (DerSimonian-Laird method) implemented in the meta package of R software. The meta-analysis encompassed seven cohorts (GSE2658, GSE24080, GSE9782, GSE136337, GSE57317, GSE4581, and MMRF, comprising a total of 3,117 patient).

### 2.5 Single-cell data collection and processing

Methods for single-cell data analysis are detailed in the **Supplementary Materials-Methods**.

### 2.6 Cells and reagents

#### 2.6.1 Cell culture

U266 and RPMI-8226 cells were purchased from the Chinese Academy of Sciences Cell Bank and identified by STR, and no mycoplasma infection detected. U266 and RPMI-8226 cells were cultured in RPMI-1640 supplemented with 10% fetal bovine serum in an incubator at 37 °C with 5% CO2 and saturated humidity.

#### 2.6.2 Reagents

RPMI-1640 and Fetal bovine serum were purchased from Basal Media (China, Fuzhou); a GoScript™ Reverse Transcription Mix, Random Primers Kit was purchased from Promega (USA, Massachusetts), TRIzol and ChamQ SYBR qPCR Master Mix (Low ROX Premixed), and DEPC-Treated Water were purchased from Vazyme (China, Nanjing). RIPA lysis buffer, skim milk powder, Phenylmethanesulfonyl fluoride (PMSF), a BCA protein quantification kit, Phosphatase and protease inhibitors, an SDS-PAGE gel preparation kit, and 5× protein lysis buffer were purchased from Beyotime Bio (China, Shanghai). Tris, glycine, SDS, and protein staining markers were obtained from Solarbio (China, Shanghai). Anhydrous ethanol and methanol were purchased from Servicebio (China, Fuzhou). Cell cycle and apoptosis detection kit from BD (USA, New York). Cell Counting Kit-8 (CCK8) and enhanced chemiluminescence (ECL) reagents were purchased from ABK BIO(China, Xiamen).

### 2.7 siRNA transfection

siRNA was designed and synthesized by Magen Biotech (Wuhan, China). U266 or RPMI-8226 cells were seeded in serum-free 1640 medium and transfected with 50 nM siRNA using Lipofectamine 2000, following manufacturer’s protocol. After 4–6 hours of incubation, the medium was replaced with complete medium. The siRNA sequences are as follows: UAP1-siRNA-1: ATGCATTATTCCATGGTATAT; UAP1-siRNA-2: CCAGACAAACCCAATGGAATA.

### 2.8 Quantitative reverse‐transcription polymerase chain reaction

Total RNA was isolated from U266 and RPMI-8226 cells using TRIzol reagent, followed by reverse transcription using a commercial cDNA synthesis kit. RT-qPCR was performed using Hieff^®^ qPCR SYBR Green Master Mix (No Rox) (Yeasen Biotechnology, Shanghai, China) on a Biosystems 7500 system. Gene expression levels were normalized to GAPDH as an internal control and quantified using the 2^−ΔΔCt^ method. The primer sequences for all target genes are listed in **Supplementary Table 0** (14).

### 2.9 CCK-8 proliferation assay

Cell proliferation was assessed by CCK-8 assay. U266 and RPMI-8226 cells were seeded in 96-well plates (5×10^3^cells/well) and cultured for 24–96 hours. After adding CCK-8 reagent, absorbaFFnce was measured at 450 nm. All assays were performed in triplicate and independently repeated. For drug sensitivity, dexamethasone was serially diluted and added to cells, and IC50 was determined from OD450 values using GraphPad after 24 hours.

### 2.10 Western-blotting

Total protein was extracted from U266 and RPMI-8226 cells using RIPA lysis buffer. The protein samples were separated by 10% SDS-PAGE and transferred onto nitrocellulose membranes. After blocking with 5% skim milk for 2 h, the membranes were incubated overnight at 4 °C with the following primary antibodies: anti-UAP1 (A8662, 1:1000, Proteintech, China) and anti-β-actin (AC026, 1:20,000, Proteintech, China). Subsequently, the membranes were probed with a horseradish peroxidase (HRP)-conjugated secondary antibody (TA-005, 1:20,000, Tagene Bio, China) for 1 h at room temperature. Protein bands were visualized using an enhanced ECL detection system.

### 2.11 Flow cytometry analysis

#### 2.11.1 Apoptosis assay

Transfected U266 and RPMI-8226 cells were harvested and subjected to apoptosis analysis using Annexin V-APC/7-AAD Apoptosis Kit. After dyeing according to the manufacturer’s protocol, samples were analyzed immediately using a BD FACS Canto II flow cytometer (BD Biosciences).

#### 2.11.2 Cell cycle analysis

For cell cycle distribution assessment, transfected cells were fixed in ice-cold 75% ethanol at -20 °C overnight. Following fixation, cells were washed with PBS and stained with propidium iodide (PI) solution containing RNase A. DNA content was then analyzed using the BD FACS Canto II flow cytometer.

#### 2.11.3 Surface marker analysis

U266 and RPMI-8226 MM cell lines were co-cultured with healthy human peripheral blood mononuclear cells (PBMCs) for 24 hours. Following incubation, cells were harvested and washed twice with PBS. For surface marker staining, cells were resuspended in PBS (50 μL/tube) containing fluorescently-labeled antibodies (0.5 μL antibody per tube) and incubated for 15 minutes at room temperature in the dark. After staining, cells were washed twice with FACS buffer (2% FBS in PBS) and finally resuspended in 400 μL of FACS buffer for flow cytometry analysis(A total of three healthy volunteers were included, and each experiment was independently repeated three times).

### 2.12 UDP-GlcNAc levels

Total protein was extracted from U266 and RPMI−8226 cells. UDP−GlcNAc levels were measured as follows. Briefly, 50 µL of each sample was mixed with 50 µL of 1 M HCl and hydrolyzed at 80 °C for 20 minutes. After cooling on ice and brief centrifugation, the hydrolysate was neutralized with 10 µL of 1 M KOH containing 200 mM potassium tetraborate, incubated at 80 °C for 25 minutes, and then chilled on ice. Subsequently, 150 µL of Ehrlich’s reagent (0.67 M p−dimethylaminobenzaldehyde and 11% (v/v) hydrochloric acid, diluted 1:2 in acetic acid immediately before use) was added, and the mixture was incubated at 37 °C for 30 minutes. Finally, the samples were centrifuged, and 200 µL of the supernatant was transferred to measure the absorbance at 595 nm. The results were calculated and normalized for analysis.

## 3 Statistics

Statistical analyses were performed using R software (version 4.2.3) and GraphPad Prism 9.0. Data from *in vitro* experiments are presented as mean ± SD. For clinical data, survival analyses included Kaplan-Meier curves with log-rank tests and Cox proportional hazards models. Group comparisons employed t-tests, ANOVA, or Fisher’s exact test, as appropriate. The optimal cutoff for UAP1 expression was determined using the surv-cutpoint function. A two-tailed P-value < 0.05 was considered statistically significant. Statistical significance was defined as a two-tailed P-value <0.05 for all analyses.

## 4 Results

### 4.1 Baseline characterisation of 3,117 patients with MM in the GEO database (GSE2658, GSE24080, GSE9782, GSE136337, GSE57317, GSE4581) and MMRF datasets

A total of seven different databases with clinical characteristics of multiple myeloma (MM) patients were collected (**Table 1**), including GSE2658 (559 patients), GSE24080 (559 patients), GSE9782 (264 patients), GSE136337 (426 patients), GSE57317 (55 patients), GSE4581 (411 patients), and MMRF (843 patients). The study population comprised 59.7% male and 40.3% female patients with a consistent sex distribution across the databases. Age stratification revealed that 47.8% of the patients were ≥ 60 years old, while 52.2% were younger than 60 years, with similar age distributions observed across the datasets. The racial composition was predominantly White/Caucasian (89.23%), with smaller proportions of Asian, Black, Hispanic, and Native American patients.

**Table 1:** Summary of patient clinical information from 7 databases.

	Overall	GSE2658	GSE24080	GSE9782	GSE136337	GSE57317	GSE4581	MMRF
Variables	3117	559	559	264	426	55	411	843
Platform.id (%)	426 (13.67)	0 (0.00)	0 (0.00)	0 (0.00)	426 (100.00)	0 (0.00)	0 (0.00)	0 (0.00)
GPL27143	1584 (50.82)	559 (100.00)	559 (100.00)	0 (0.00)	0 (0.00)	55 (100.00)	411 (100.00)	0 (0.00)
GPL570	264 (8.47)	0 (0.00)	0 (0.00)	264 (100.00)	0 (0.00)	0 (0.00)	0 (0.00)	0 (0.00)
GPL96	843 (27.05)	0 (0.00)	0 (0.00)	0 (0.00)	0 (0.00)	0 (0.00)	0 (0.00)	843 (100.00)
MMRF								
Gender (%)								
Female	843 (40.30)	0 (NaN)	222 (39.71)	105 (39.77)	165 (38.73)	0 (NaN)	0 (NaN)	351 (41.64)
Male	1249 (59.70)	0 (NaN)	337 (60.29)	159 (60.23)	261 (61.27)	0 (NaN)	0 (NaN)	492 (58.36)
Age (%)								
<60	652 (52.20)	0 (NaN)	318 (56.89)	121 (45.83)	213 (50.00)	0 (NaN)	0 (NaN)	0 (NaN)
>=60	597 (47.80)	0 (NaN)	241 (43.11)	143 (54.17)	213 (50.00)	0 (NaN)	0 (NaN)	0 (NaN)
Race (%)								
Asian	17 (1.06)	0 (NaN)	0 (0.00)	0 (NaN)	4 (0.94)	0 (NaN)	0 (NaN)	13 (1.90)
Black	147 (9.15)	0 (NaN)	0 (0.00)	0 (NaN)	35 (8.24)	0 (NaN)	0 (NaN)	112 (16.35)
Hispanic	6 (0.37)	0 (NaN)	0 (0.00)	0 (NaN)	6 (1.41)	0 (NaN)	0 (NaN)	0 (0.00)
Native American	3 (0.19)	0 (NaN)	0 (0.00)	0 (NaN)	3 (0.71)	0 (NaN)	0 (NaN)	0 (0.00)
White/Caucasian	1434 (89.23)	0 (NaN)	497 (100.00)	0 (NaN)	377 (88.71)	0 (NaN)	0 (NaN)	560 (81.75)
Iss (%)								
I	463 (47.15)	0 (NaN)	295 (52.87)	0 (NaN)	168 (39.62)	0 (NaN)	0 (NaN)	0 (NaN)
II	279 (28.41)	0 (NaN)	144 (25.81)	0 (NaN)	135 (31.84)	0 (NaN)	0 (NaN)	0 (NaN)
III	240 (24.44)	0 (NaN)	119 (21.33)	0 (NaN)	121 (28.54)	0 (NaN)	0 (NaN)	0 (NaN)
Issr (%)								
I	83 (19.81)	0 (NaN)	0 (NaN)	0 (NaN)	83 (19.81)	0 (NaN)	0 (NaN)	0 (NaN)
II	270 (64.44)	0 (NaN)	0 (NaN)	0 (NaN)	270 (64.44)	0 (NaN)	0 (NaN)	0 (NaN)
III	66 (15.75)	0 (NaN)	0 (NaN)	0 (NaN)	66 (15.75)	0 (NaN)	0 (NaN)	0 (NaN)
Stage (%)								
I	281 (34.18)	0 (NaN)	0 (NaN)	0 (NaN)	0 (NaN)	0 (NaN)	0 (NaN)	281 (34.18)
II	298 (36.25)	0 (NaN)	0 (NaN)	0 (NaN)	0 (NaN)	0 (NaN)	0 (NaN)	298 (36.25)
III	243 (29.56)	0 (NaN)	0 (NaN)	0 (NaN)	0 (NaN)	0 (NaN)	0 (NaN)	243 (29.56)
Os.status (%)								
Alive	2164 (69.43)	459 (82.11)	387 (69.23)	108 (40.91)	183 (42.96)	43 (78.18)	331 (80.54)	653 (77.46)
Dead	953 (30.57)	100 (17.89)	172 (30.77)	156 (59.09)	243 (57.04)	12 (21.82)	80 (19.46)	190 (22.54)

The number 0 indicates that no corresponding data was found.

Disease staging and survival analysis: The International Staging System (ISS) classification revealed the following distribution: stage I (47.15%), stage II (28.41%), and stage III (24.44%), with some inter-dataset variability observed in stages II and III. Notably, the immunity subtype-specific risk stratification showed a distinct pattern, with stage II comprising the majority (64.44%) of cases, followed by stage III (15.75%). Regarding clinical outcomes, the overall cohort demonstrated a survival rate of 69.43% (30.57% mortality), with minor variations across different datasets.

### 4.2 UAP1 expression may serve as a prognostic biomarker in MM

**Figure 1:**
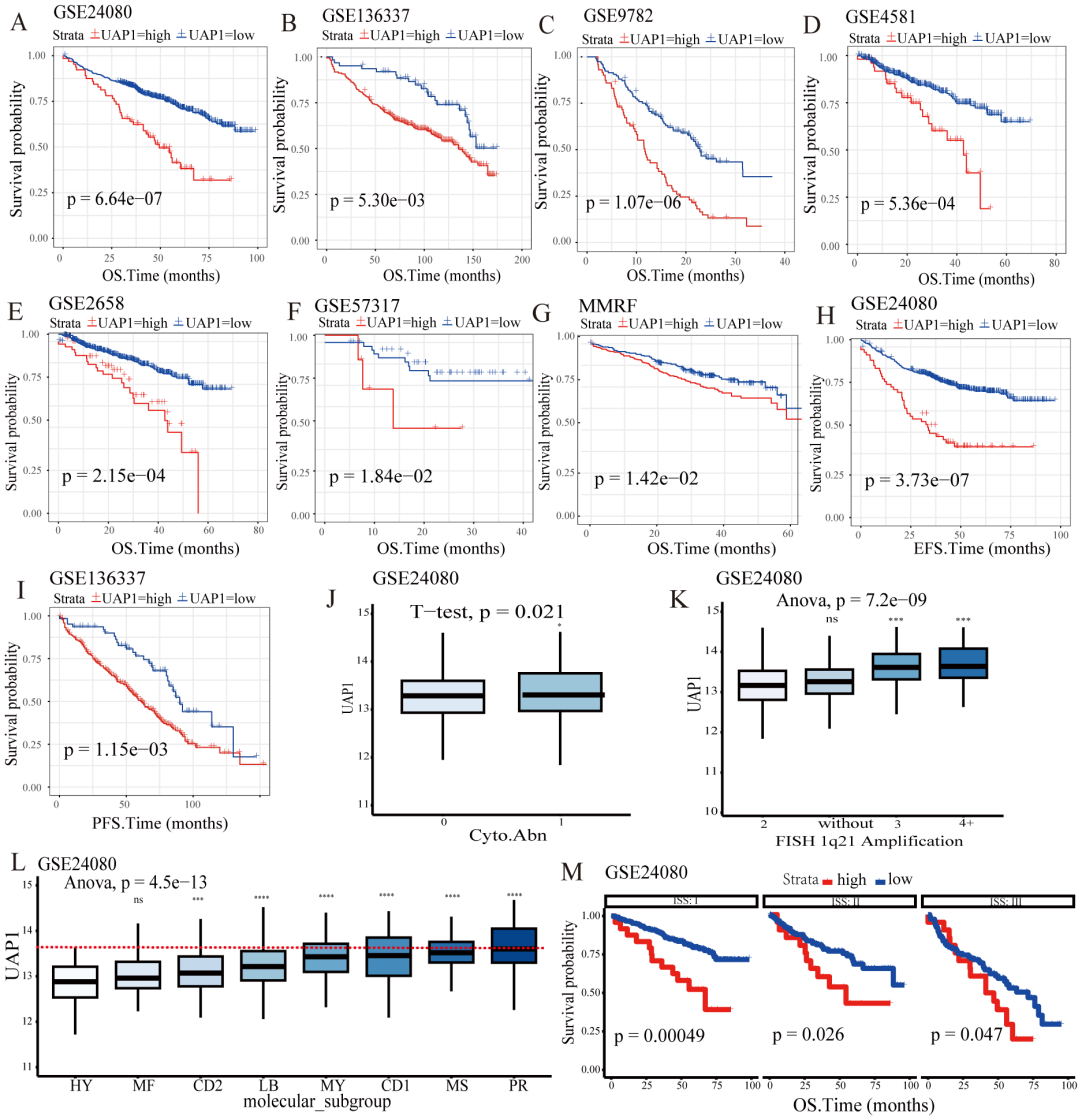
Correlation between UAP1 expression levels and prognosis in multiple myeloma. **(A–G)** Correlation of UAP1 expression levels with OS in seven datasets; **(H, I)** Correlation of UAP1 expression with EFS in GSE24080 **(H)** and with PFS in GSE136337 **(I)**; **(J)** Association of UAP1 expression with cytogenetic abnormalities; **(K)** Association with FISH-detected 1q21 high amplification. **(L)** Association with molecular subgroups; **(M)** Association with OS stratified by disease stage. (OS, Overall Survival; EFS, Event-Free Survival; PFS, Progression-Free Survival; EFS and PFS data were available only in the two indicated datasets (GSE24080 and GSE136337).

Accumulating evidence from prior studies indicates that UAP1 functions as both a prognostic biomarker and a promising therapeutic target across diverse malignancies, including prostate cancer, bladder cancer, lung adenocarcinoma, thyroid cancer, and osteosarcoma (13, 14, 17, 18, 20). Therefore, we evaluated the prognostic significance of UAP1 expression in MM by analyzing the Kaplan–Meier survival curves for OS, EFS, and PFS across multiple independent datasets. Patients were stratified into high- and low-expression groups based on their UAP1 expression levels, and between-group comparisons were performed using log-rank tests. Consistent with its potential role as a negative prognostic factor, elevated UAP1 expression was significantly associated with poorer survival outcomes in all seven analyzed datasets (GSE24080, GSE136337, GSE9782, GSE4581, GSE2658, GSE57317, and MMRF; all *P* < 0.05; **Figures 1A–G**). Notably, In the GSE24080 cohort, high UAP1 expression correlated with significantly shorter EFS (*P* = 3.73 × 10-7; **Figure 1H**). The GSE136337 dataset demonstrated superior PFS in the low-expression group compared with the high-expression group (*P* = 1.15 × 10-3; **Figure 1I**).

### 4.3 Association analysis between UAP1 expression levels and clinical characteristics, genetic profiles, and molecular abnormalities in patients with MM

To assess the relationship between UAP1 expression and clinical features of MM, we categorized 559 patients (GSE24080) into high- and low-expression groups based on UAP1 levels and performed comparative analyses of their molecular and clinical characteristics (**Supplementary Table 1**). No significant differences were observed between the two groups in demographic characteristics (race, sex, age) or laboratory parameters including Beta-2 microglobulin (β2M), Lactate dehydrogenase (LDH), Creatinine (CREAT), C-reactive protein (CRP), Albumin (ALB), Hemoglobin (HGB), Bone marrow plasma cells (BMPC), and Magnetic resonance imaging (MRI) findings. However, a statistically significant difference was noted in ISS staging distribution (*P* = 0.0248). Furthermore, subgroup analyses revealed significant differences between the two groups in the distribution of UAMS molecular classification subtypes (CD1, CD2, HY, LB, MF, MS, MY, and PR; *P* = 0.0004). Cytogenetic analysis revealed two key findings: First, the UAP1 high expression group harbored significantly more genetic aberrations (*P* = 0.0001); second, this group showed enrichment for FISH 1q21 amplification (*P* < 0.0001), particularly in the 3+-copy and 4+-copy variant subgroup known to confer poor outcomes. This strong association between UAP1 overexpression and high-risk genetic features suggests a potential role in MM progression.

### 4.4 Poorer clinical characteristics are consistently associated with higher UAP1 expression

To further investigate the relationship between molecular abnormalities and UAP1 expression in patients with MM, we analyzed the cytogenetic and molecular subgroups. Notably, patients harboring cytogenetic abnormalities exhibited significantly elevated UAP1 expression (*P* = 0.021; **Figure 1J**). ANOVA analysis further revealed that UAP1 expression was markedly higher in the FISH 1q21 highly amplified group (3+, 4+) compared to other subgroups (*P* = 7.2e−09; **Figure 1K**). Significant differences in UAP1 levels were observed across multiple molecular subgroups, with the highest expression detected in the PR subgroup (**Figure 1L**). Most importantly, Kaplan–Meier survival analysis demonstrated that patients with MM with high UAP1 expression had significantly poorer outcomes than those in the low-expression group, independent of ISS stage (**Figure 1M**). Collectively, these findings underscore the association between UAP1 expression and adverse clinical features and high-risk molecular subtypes, suggesting its potential role as a prognostic marker for MM.

### 4.5 UAP1 serves as an independent prognostic factor in MM

To comprehensively evaluate the prognostic value of UAP1 in MM, we conducted univariate and multivariate Cox regression analyses of OS and EFS using the GSE24080 dataset (n = 559). The following clinical and molecular variables were included: Demographics: Age (<60 vs. ≥60 years), sex (female vs. male), ethnicity (White vs. other); Laboratory parameters: ALB (low vs. high), β2M (low vs. high), BMPC (low vs. high), Aspartyl Protease C (low vs. high), CREAT (low vs. high), CRP (low vs. high), HGB (low vs. high), LDH (low vs. high); Disease characteristics: Immunoglobulin type (FLC vs. IgA vs. IgD vs. IgG vs. nonsecretory), ISS stage (I vs. II vs. III), cytogenetic abnormalities (Cyto.Abn, 0 vs. 1), FISH 1q21 amplification (NA vs. 2 vs. 3 vs. 4+), MRI status (low vs. high); Molecular subgroups: MM molecular subtypes (CD1, CD2, HY, LB, MF, MS, MY, PR); and UAP1 gene expression level (low vs. high). Each variable was assessed using univariate Cox regression for OS and EFS, followed by multivariate analysis to determine independent prognostic factors.

Univariate Cox regression analysis revealed several significant prognostic factors: Patients <60 years showed significantly worse OS (HR = 1.42, *P* = 0.023) compared to ≥60 years, though no significant difference was observed for EFS (p=0.141). Low ALB expression was strongly associated with poor OS (hazard ratio [HR] = 0.482, *P* = 2.46E-06) and EFS (HR = 0.599, *P* = 5.61E-05). Elevated β2M expression significantly correlated with adverse prognosis for both OS (HR = 2.3, *P* = 2.38E-07) and EFS (HR = 2.01, *P* = 9.67E-08). Cytogenetic abnormalities were significantly associated with worse survival outcomes for both OS (HR = 2.29, *P* = 6.01E-08) and EFS (HR = 1.79, *P* = 6.00E-04)).

In the multivariate analysis, low ALB expression remained significantly associated with worse OS (HR = 0.51, *P* = 1.00E-04), although its impact on EFS was attenuated (*P* = 0.216). Elevated β2M levels were significantly associated with EFS (HR = 1.56, *P* = 0.0014). Although a trend was observed, its effect on OS was not statistically significant (HR = 1.32, *P* = 0.255). Cytogenetic abnormalities demonstrated independent prognostic value for OS (HR = 1.79, 95% CI [1.26-2.55], *P* = 0.0014) but not for EFS (*P* = 0.164). High LDH expression emerged as a strong independent predictor for both OS (HR = 1.61, 95% CI [1.21-2.14], *P* = 8.00×10^−4^) and EFS (HR = 1.42, 95% CI [1.15-1.76], *P* = 0.0014). Most notably, high UAP1 expression demonstrated consistent and significant associations across univariate analyses (OS (HR = 2.47, *P* = 1.53e-06) and EFS (HR = 2.03, *P* = 6.0e-04)) and multivariate models (OS (HR = 2.22, *P* = 1.75e-06) and EFS (HR = 1.78, *P* = 0.0017)). These robust findings confirmed that UAP1 is an independent prognostic marker in MM (**Supplementary Table 2**).

### 4.6 Validation of UAP1 as an independent prognostic factor in the GSE136337 cohort

To further validate the potential value of UAP1 in prognostic prediction of patients with MM, we utilised the GSE136337 dataset of 426 MM patients for further validation. We analysed baseline characteristics (**Supplementary Table 3**) as well as univariate and multivariate Cox regression analyses for OS and EFS (**Supplementary Table 4**). Regarding OS, the proportion of deaths was higher in the UAP1 high expression group (*P* = 0.015). For ISS staging, the proportion of stage III patients was significantly higher in the UAP1 high-expression group, and these results suggest that high UAP1 expression was associated with a poorer prognosis. In addition, the distribution of gender, age, and race did not show significant differences between the two groups. At the ALB level, the difference between the two groups was statistically significant (*P* = 0.0015). Finally, subgroup analysis revealed that high UAP1 expression was associated with multiple subgroups, suggesting that UAP1 expression may be closely linked to various clinical characteristics.

Univariate analyses demonstrated that patients younger than 60 years experienced significantly better OS (HR = 1.64, *P* = 0.000949) and EFS (HR = 1.35, *P* = 0.0264) outcomes. Age remained an independent prognostic factor for OS in multivariate analysis (HR = 1.28, *P* = 0.0024). While low ALB levels were significantly associated with worse OS (HR = 0.632, *P* = 0.00209) and EFS (HR = 0.651, *P* = 0.00133) in the univariate analysis, this association did not retain statistical significance for EFS in the multivariate context. Gender had no significant impact on either OS or EFS (all *P* > 0.05).

The ISS and R-ISS staging systems exhibited significant correlations with both OS (ISS: HR = 1.66, *P* = 4.58E-08; R-ISS: HR = 1.5, *P* = 0.00147) and EFS (ISS: HR = 1.55, *P* = 7.69E-08; R-ISS: HR = 1.52 *P* = 0.000129) in the univariate analysis. However, only ISS staging retained independent prognostic significance in the multivariate models (OS (HR = 1.81, *P* = 4.5E-10); EFS (HR = 1.53, *P* = 9.00E-04)). Subgroup analyses revealed no statistically significant differences in survival outcomes across the examined strata (all *P* > 0.05). Notably, and consistent with findings from the GSE24080 dataset, elevated UAP1 expression was robustly associated with inferior survival outcomes in both univariate and multivariate analyses (OS and EFS: All HR > 1, all *P* < 0.01). Collectively, these results confirm that high UAP1 expression serves as a robust, independent predictor of poor prognosis in MM.

We also performed a meta-analysis across multiple cohorts, with the results presented as a forest plot. The analysis revealed no significant heterogeneity among the cohorts (τ² = 0.0012; Chi² = 5.83, df = 6, P = 0.4427, I² ≈ 0%), and the effect directions were highly consistent. The pooled HR was approximately 2.3, with a 95% confidence interval that did not cross 1. Furthermore, the association between high UAP1 expression and poor overall survival was robust and reproducible across multiple independent cohorts (**Supplementary Figure 1**).

### 4.7 UAP1 exhibits tumor-specific overexpression and participates in the differentiation of distinct tumor cell subpopulations

**Figure 2:**
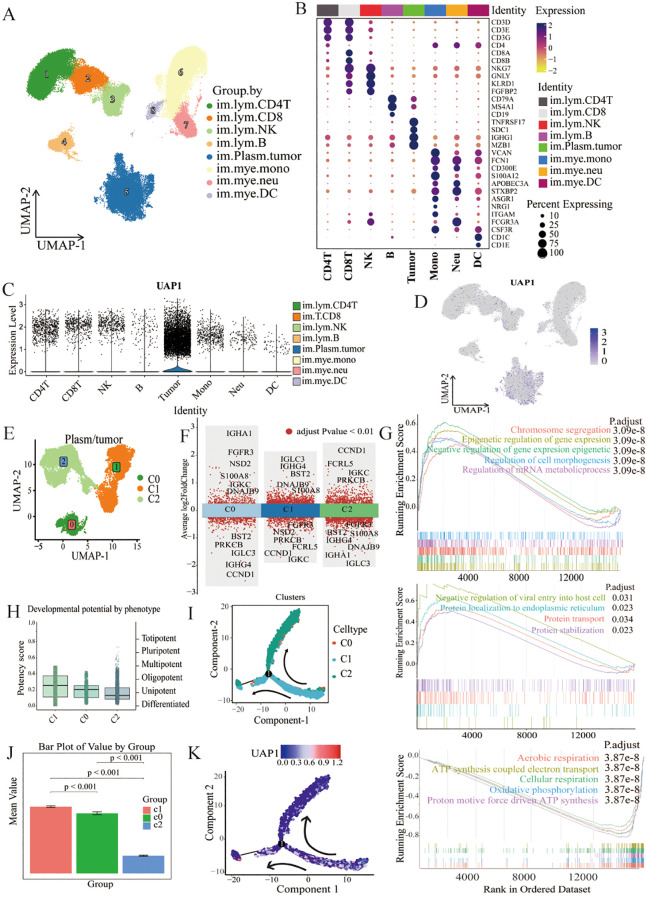
Elevated UAP1 expression in tumor cells and its association with cellular differentiation status. **(A)** UMAP visualization of single-cell transcriptomic data; **(B)** Cell type-specific gene expression profiles across distinct clusters. **(C)** Scatter plot showing the distribution of UAP1 expression levels. **(D)** UMAP projection of UAP1 expression across different cell populations. **(E)** Identification of tumor cell subpopulations via UMAP clustering. **(F)** Volcano plot of differentially expressed genes among tumor subtypes. **(G)** Gene set enrichment analysis (GSEA) of enriched pathways among tumor subtypes. **(H)** Developmental potential scores of C0, C1, and C2. **(I)** Pseudotime trajectory analysis (based on PCA dimensionality reduction) with cell type annotations. **(J)** UAP1 expression levels in C0, C1, and C2 tumor subpopulations. **(K)** Correlation analysis between developmental potential and UAP1 expression. (DC, Dendritic Cell; B, B Cell; CD4T, CD4^+^ T Cell; NK, Natural Killer Cell; Mono, Monocyte; Neu, Neutrophil; CD8T, Cytotoxic T Cell).

To further investigate the specific mechanisms by which UAP1 contributes to poor prognosis in MM, we performed single-cell RNA sequencing analysis on samples derived from 24 MM cases. Following comprehensive clustering and annotation of over 70,000 single cells, we identified eight distinct immune cell populations: CD4^+^ T cells, CD8^+^ T cells, natural killer (NK) cells, B cells, plasma/tumor cells, monocytes, neutrophils, and dendritic cells (DCs). Each cell population exhibited characteristic marker gene expression patterns: CD4^+^ T cells (CD3D, CD3E, CD3G, CD4); CD8^+^ T cells (CD3D, CD3E, CD3G, CD8A, CD8B); NK cells (MKI67, GNLY, KLRD1, FGFBP2); B cells (CD79A, MS4A1, CD19); plasma/tumor cells (TNFRSF17, SDC1, IGHG1, GZMB); monocytes (VCAN, FCN1, CD300E, S100A12); neutrophils (FCGR3A); and dendritic cells (CSF3R, CD1C, CD1E) (**Figures 2A, B**; **Supplementary Figures 2A, B**; **Supplementary Tables 5–S6**). Subsequent analyses focused on characterizing the differential expression patterns of UAP1 across these cell subsets. Our data revealed that UAP1 expression was most abundant in plasma/tumor cells, with transcript levels significantly elevated in this population relative to all other immune cell types (**Figures 2C, D**).

To investigate the functional role of UAP1 within distinct tumor cell subpopulations, we performed clustering and pseudotime trajectory analysis on the identified plasma/tumor cell compartment. Three transcriptionally distinct clusters were resolved: Cluster C0 was characterized by specific high expression of *IGHA1* and *FGFR3*, which are associated with plasma cell differentiation and FGFR3−driven oncogenic signaling in MM; Cluster C1 exhibited elevated expression of *IGLC3*, *IGHG4*, and *BST2*, the latter being a marker of plasma cell activation and immune regulation; and Cluster C2 displayed prominent expression of *IGLC3* and *IGHG4*, indicating a distinct immunoglobulin−related transcriptional program. The encoded proteins of these genes are predominantly involved in cellular immune regulation, differentiation, and proliferation (**Figure 2E**). Combined analysis of the gene expression characteristics of the three cell clusters, revealed that these genes are associated with chromosome segregation, cell morphogenesis, mRNA metabolism, protein transcription, and energy supply (**Figures 2F, G**; **Supplementary Tables 7–S10**), suggesting that these three clusters of cells are functionally active and have high energy demands. These features, which are characteristic of tumor cells, further confirm that the three clusters are indeed MM cells.

Comparative analysis of differentiation potential indicated that Cluster C1 possessed the highest developmental potency and was therefore designated as the root node for trajectory inference (**Figure 2H**). The resulting differentiation trajectory revealed that C1 has the capacity to give rise to both C0 and C2, a process accompanied by a progressive downregulation of UAP1 expression (**Figures 2I–K**; **Supplementary Figures 2C–E**). Thus, C0, C1, and C2 reflect functional heterogeneity and differentiation states within the MM tumor compartment. Moreover, UAP1 expression levels were found to be associated with the kinetics of temporal differentiation and the timing of inflection point transitions along the pseudotime axis (**Supplementary Figures 2F, G**). Collectively, these findings highlight a correlative link between UAP1 and subtype−specific differentiation.

### 4.8 The expression level of UAP1 is closely related to immunosuppression

**Figure 3:**
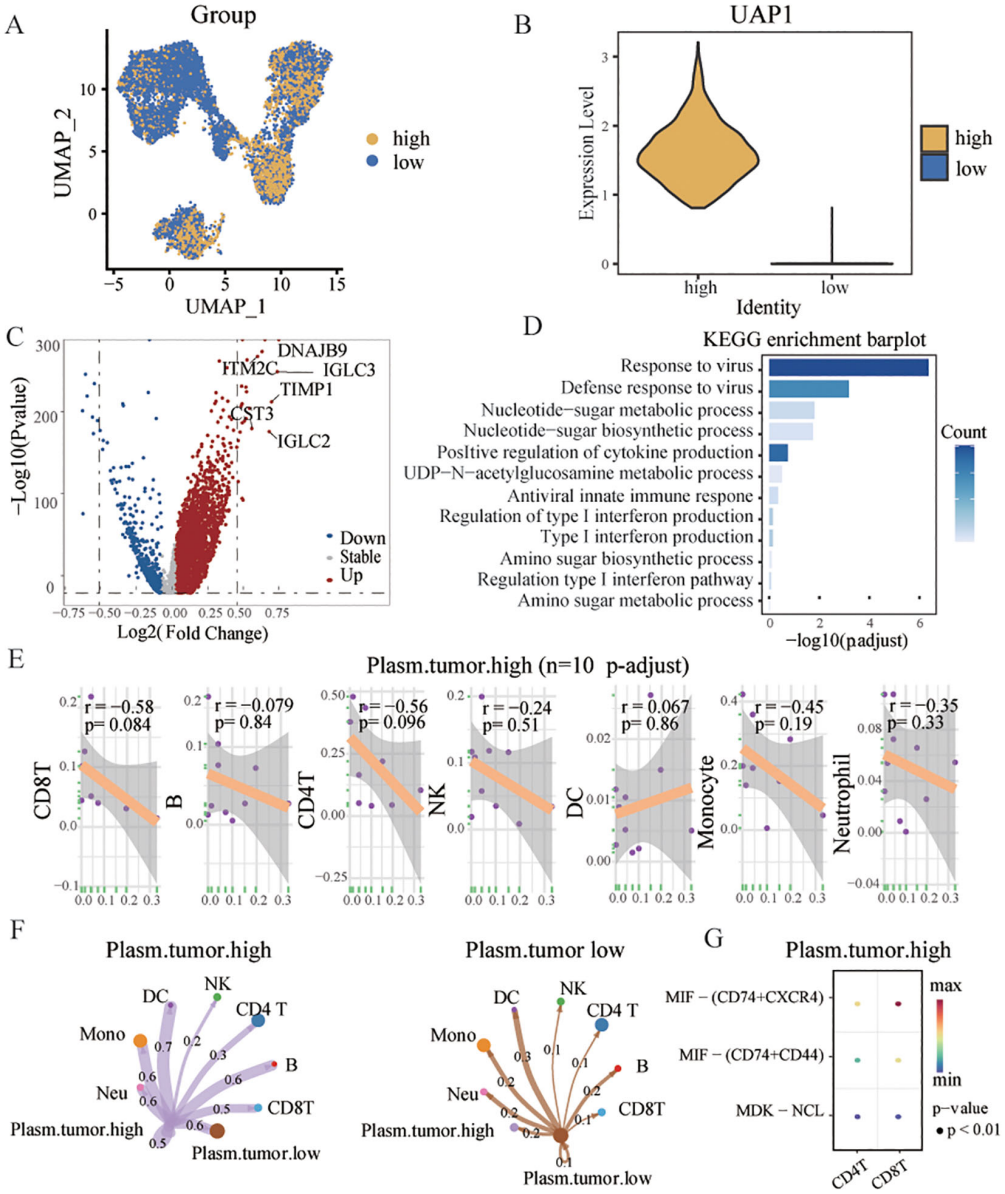
Association between high-UAP1 tumor cells and the immune microenvironment. **(A)** UMAP dimensionality reduction analysis; **(B)** Violin plots of UAP1 expression levels; **(C)** Volcano plot of differentially expressed genes between UAP1-high and UAP1-low groups; **(D)** Bar graphs of KEGG pathway enrichment; **(E)** Correlation between cell type proportions and the prevalence of UAP1-high tumor cells; **(F)** Cellular communication networks in high- versus low-UAP1 tumor cells; **(G)** Ligand-receptor interaction analysis.

MM, an immune-related hematological malignancy, is characteristically associated with a dysregulated immune microenvironment. To investigate the functional significance of UAP1-high tumor cells and their potential impact on the immune landscape, we stratified the tumor cell population into UAP1-high and UAP1-low expression subgroups using the K-means clustering algorithm. Subsequent differential gene expression analysis revealed that the UAP1-high group specifically exhibited elevated transcript levels of genes including *DNAJB9*, *ITM2C*, *IGLC3*, and *TIMP1* (**Figures 3A–D**; **Supplementary Tables 11-S12**), which encode proteins implicated in cellular immune regulation, proliferation, and metabolic homeostasis.

Analysis of immune cell proportions demonstrated that, with the exception of DCs, all other examined immune populations—CD4^+^ T cells, CD8^+^ T cells, B cells, NK cells, monocytes, and neutrophils—were present at reduced relative frequencies in samples enriched for UAP1-high tumor cells (**Figure 3E**; **Supplementary Table 13**). This inverse association was particularly pronounced for T lymphocytes (CD4^+^ and CD8^+^) and B cells, suggesting a potential link between UAP1-high tumor cells and an immunosuppressive state within the MM bone marrow microenvironment. Cell–cell communication analysis further revealed that the interaction networks emanating from UAP1-high tumor cells were significantly more robust compared to those from their UAP1-low counterparts (**Figure 3F**; **Supplementary Figures 3A–G**). Notably, the inferred interaction strength with CD8^+^ T cells (0.5) exceeded that observed with CD4^+^ T cells (0.3). Further interrogation of ligand–receptor signaling identified enhanced activation of the macrophage migration inhibitory factor (MIF) pathway, specifically mediated by MIF–(CD74^+^ CXCR4) and MIF–(CD74^+^ CD44) cognate pairs, within the interactions between UAP1-high tumor cells and both CD4^+^ and CD8^+^ T cell subsets (**Figure 3G**). The MIF pathway is a well−known regulator of immune cell trafficking and activation. In MM, MIF has been reported to promote tumor progression and suppress T cell effector functions. Our observation that UAP1 expression correlates with MIF pathway activity (as shown in **Figure 3F**) provides a mechanistic link: UAP1 may modulate the MIF signaling axis to alter CD8^+^ T cell infiltration and function.

### 4.9 UAP1 drives malignant progression and modulates immune cell infiltration, representing a promising therapeutic target for MM

**Figure 4:**
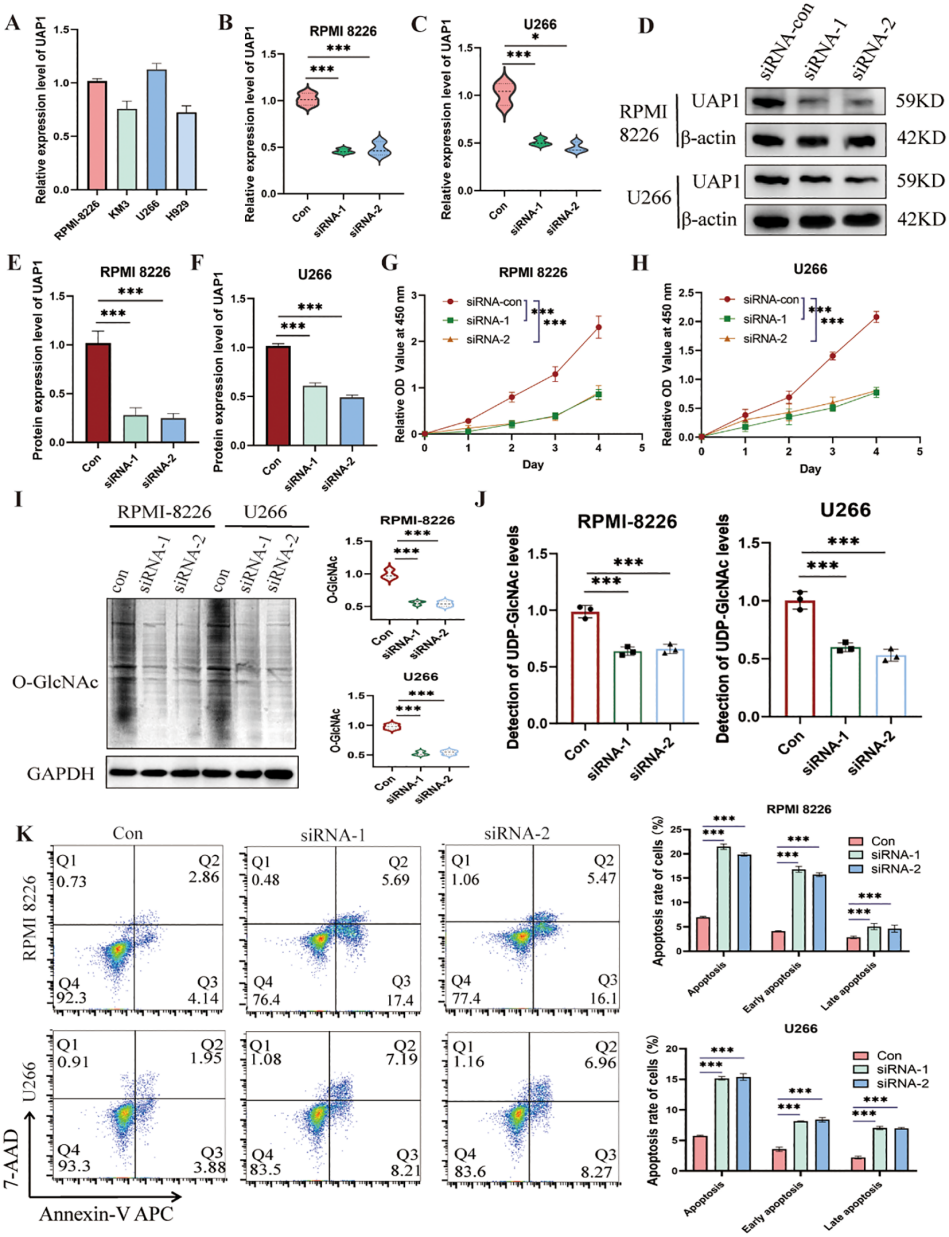
UAP1 regulates proliferation and apoptosis in multiple myeloma cells. **(A)** Expression levels of UAP1 genes in RPMI-8226, U266, H929 and KM3 cells; **(B–F)** UAP1 knockdown efficiency was confirmed at both mRNA and protein levels using RT-qPCR and Western blotting, respectively; **(G, H)** RPMI-8226 and U266 cell proliferation were detected using CCK8 assay; **(I)** O-GlcNAcylated protein levels were detected by Western blotting; **(J)** Detection of UDP-GlcNAc levels; **(K)** RPMI-8226 and U266 cell apoptosis rates were detected using flow cytometry. (^*^P < 0.05, ^**^P < 0.01, ^***^P < 0.001; All experiments were repeated 3 times; The gel image in **(D)** was cropped from different lanes of the same gel).

These collective findings indicate that UAP1 may serve as a novel and independent prognostic biomarker in MM. While also playing a critical role in regulating both tumor cell differentiation programs and immunosuppressive microenvironment remodeling. Prompted by these observations, we next explored the potential of UAP1 as a therapeutic target for MM using *in vitro* cellular assays. Two MM cell lines exhibiting high basal UAP1 expression, RPMI−8226 and U266, were selected for subsequent investigations (**Figure 4A**). To directly assess the functional consequences of UAP1 depletion, we performed siRNA−mediated knockdown of UAP1 in both cell lines. The qPCR and protein analyses confirmed effective suppression of UAP1 at both the transcript and protein levels (**Figures 4B–F**). Following UAP1 knockdown, cell proliferation was markedly attenuated in both RPMI−8226 and U266 cells (**Figures 4G, H**). The levels of O-GlcNAcylated proteins and UDP-GlcNAc were significantly reduced upon UAP1 knockdown (**Figures 4I, J**), which is consistent with the known functional role of UAP1. However, MIF expression levels were not affected by UAP1 knockdown (**Supplementary Figure 3H**). Concurrently, apoptosis was significantly enhanced in the knockdown group compared to controls, with early apoptosis being the predominant form (**Figure 4K**).

**Figure 5:**
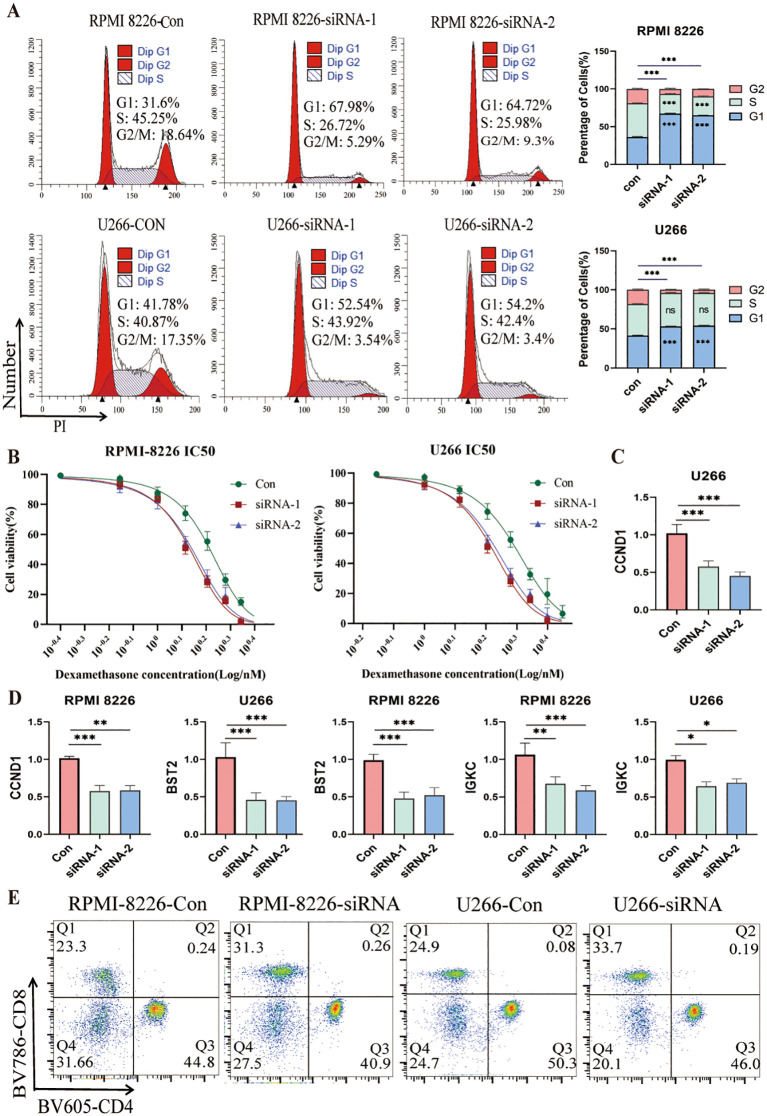
UAP1 regulates cell cycle progression, drug sensitivity, and he proportion of CD8+ T cells in multiple myeloma. **(A)** Flow cytometric analysis of cell cycle distribution in RPMI-8226 and U266 cells following UAP1 knockdown; **(B)** Sensitivity of RPMI-8226 and U266 cells to dexamethasone detected after UAP1 knockdown; **(C, D)** mRNA expression levels of CCND1, IGKC, and BST2 genes after UAP1 gene knockdown; **(E)** Flow cytometric analysis of immune cell subsets in co-cultures of UAP1-knockdown RPMI-8226 or U266 cells with healthy donor PBMCs. (^*^P < 0.05, ^**^P < 0.01, ^***^P < 0.001; All experiments were repeated 3 times).

Additionally, we assessed cell cycle changes following UAP1 knockdown. Both MM cell lines exhibited a pronounced accumulation of cells in the G1 phase relative to the control group, a finding consistent with the observed suppression of cell proliferation (**Figure 5A**). Finally, drug sensitivity (**Figure 5B**) assays demonstrated that UAP1 knockdown significantly enhanced dexamethasone sensitivity in both RPMI-8226 and U266 cell lines, as evidenced by a marked reduction in IC_50_ values (RPMI-8226: 43.93nM to 22.92/24.48nM, U266: 95.34nM to 45.22/50.59nM).

Our single-cell analyses revealed that UAP1 expression is significantly associated with key cellular processes, including proliferation, differentiation, and immune regulation. Based on biological function and communication analysis, we prioritized the top five candidate genes from **Figure 2F** (*CCND1*, *IGHG4*, *IGLC3*, *IGKC* and *BST2*) for further experimental validation. Upon UAP1 knockdown, the expression levels of *CCND1*, *IGKC* and *BST2* were significantly downregulated (**Figures 5C, D**). Given the established roles of these genes in multiple myeloma cell proliferation, malignant progression, and immune modulation, these findings further corroborated the reliability and consistency of our preceding studies. However, no significant differences were found in the *IGHG4* and *IGLC3* genes (**Supplementary Figures 3I, J**). These data indicated that UAP1 modulates the differentiation of MM cells by regulating key molecules within tumor cells, thereby mediating immunosuppression in the tumor microenvironment.

Notably, our prior findings demonstrated that UAP1−high tumor cells exhibit immunosuppressive properties, characterized by particularly robust interactions with CD8^+^ T cells (Result 4.8). To experimentally validate these observations, we established a co-culture system using two MM cell lines (with UAP1 knockdown constructs) and PBMCs isolated from healthy donors. Strikingly, UAP1 knockdown resulted in a significant increase in CD8^+^ T cell proportions (*P* < 0.05), thereby providing functional confirmation of the immunosuppressive role of UAP1 in MM (**Figure 5E**; **Supplementary Figure 3K**). These data strongly suggested that UAP1-mediated immune evasion may represent a key mechanism driving tumor progression in MM.

Collectively, our results demonstrate that UAP1 plays an important role in regulating tumour cell differentiation, proliferation, and modulating immune cell function. These findings establish UAP1 as both an prognostic biomarker Regulating Malignant Biological Functions in Multiple Myeloma Cells.

## 5 Discussion

MM is a hematological malignancy, representing approximately 1.3% of all cancers and 15% of hematological neoplasms, with an estimated annual incidence of 4.5–6 cases per 100,000 individuals (21). Over the past decade, significant advancements in treatment have led to marked improvements in patient prognosis and survival rates (22, 23).Nevertheless, MM remains an incurable disease characterized by considerable biological heterogeneity (24). Despite extensive research having been conducted, the underlying etiology of MM remains unclear. Consequently, the identification of novel biomarkers is critical for enhancing therapeutic strategies and prognostic assessments in patients with MM.

Previous studies have shown that UAP1 regulates the N-strand glycosylation levels of cancer cells by affecting HBP and promoting cancer cell proliferation and migration (25, 26). In this study, we found that UAP1 is a meaningful predictor of MM prognosis. Among them, we analyzed 3,117 patients and for the first time identified UAP1 as a prognostic indicator of poor outcomes and a potential therapeutic target in patients with MM. Patients with MM and high UAP1 expression demonstrated significantly reduced OS and EFS, a finding that was further validated using independent datasets. Our study further corroborates the findings of Luis et al. (27), who demonstrated that UAP1 serves as a marker in MM. More importantly, our investigation represents the first large-scale analysis encompassing data from 3,117 patients, thereby substantiating the reliability of UAP1 as a prognostic indicator in MM.

In contrast, our study revealed that patients with high UAP1 expression exhibited poorer OS and PFS, while UAP1 knockdown enhanced dexamethasone sensitivity. Furthermore, the expression level of UAP1 showed high consistency with both current molecular subtypes and clinical staging of MM, a clonal B-cell malignancy characterized by excessive abnormal bone marrow plasma cells (28). Abnormalities in Cyto.Abn are known to indicate poor prognosis in MM patients. In this study, we observed a significantly higher percentage of Cyto.Abn in patients with elevated UAP1 expression. The development and accumulation of various combinations of chromosomal and molecular abnormalities are well-established factors that elevate the risk of adverse outcomes in MM (29, 30).

Notably, tumorigenesis and drug resistance are partially mediated by upregulation of genes located on the 1q21 amplicon, and patients with this amplification exhibit worse prognosis than those without it (31). Our study demonstrated that MM patients harboring 3+ and 4+ copies of the chromosomal 1q21 amplicon (detected by FISH) consistently showed elevated UAP1 expression. The University of Arkansas for Medical Sciences (UAMS) myeloma group has established a widely recognized molecular classification system based on MM cytogenetic abnormalities, categorizing MM into seven distinct molecular subgroups: CD-1, CD-2, MS, MF, LB, HY, and PR groups (32). Previous findings have identified the MF and PR subgroups as highest-risk, followed by the MS and MY subgroups, while the CD1, CD2, HY and LB subgroups were classified as lowest-risk standard groups (33). Importantly, our study further confirmed that UAP1 expression was most prominent in the PR group and showed distinct differential expression patterns across all subgroups. In addition, the International Myeloma Working Group (IMWG) established ISS staging based on two common clinical indicators (serum β2-microglobulin and albumin levels), and subsequently developed R-ISS staging by incorporating LDH levels and high-risk cytogenetic abnormalities including del (17p), t (4;14), and t (14;16) detected by FISH (34). Our study demonstrated that high UAP1 expression was associated with significantly lower OS across ISS stages. Furthermore, the current study revealed that elevated UAP1 expression correlated with traditional diagnostic markers including albumin and absolute serum plasma cell (ASPC) levels (35). These findings collectively support the potential utility of UAP1 as both a diagnostic and prognostic biomarker for MM patients.

Moreover, to elucidate the precise mechanisms underlying the association between UAP1 expression and poor prognosis in patients with MM, we analyzed single−cell RNA sequencing data from 24 MM cases. Our analysis revealed that UAP1 is predominantly upregulated in malignant plasma cells, with particularly elevated transcript levels observed in distinct tumor subpopulations. Notably, we identified multiple distinct cell types, including DCs, CD4^+^ T cells, CD8^+^ T cells, B cells, NK cells, monocytes, and neutrophils. Among these populations, except for DCs, the relative proportions of all immune cell subsets exhibited an inverse correlation with UAP1 expression levels in tumor cells, suggesting that UAP1 may modulate MM cell function to facilitate immune evasion. This observation is consistent with recent studies demonstrating that the bone marrow microenvironment of patients with MM is characterized by an immunosuppressive state (36, 37).

Furthermore, we identified three distinct MM tumor cell subpopulations (designated C0, C1, and C2) that differ in their differentiation status and exhibit functional properties correlated with UAP1 expression. Specifically, the C1 cluster displayed features resembling those of tumor stem−like cells, possessed the highest functional activity, and exhibited the capacity to differentiate into the C0 and C2 clusters. Notably, UAP1 expression was most elevated in the C1 subpopulation. From the gene signatures defining these three clusters, we selected the top five candidate genes (CCND1, IGHG4, IGLC3, IGKC, and BST2) for *in vitro* validation. Following siRNA−mediated knockdown of UAP1, the expression levels of CCND1, IGKC, and BST2 were significantly suppressed. These genes have previously been implicated in the regulation of tumor cell metabolism and immune function (38–41), these findings may be related to the observed decrease in immune cell proportions.

Additionally, pathway enrichment analysis of differentially expressed genes between the UAP1−high and UAP1−low groups revealed significant enrichment of metabolic and immune−related pathways. Further analysis demonstrated that UAP1−high MM cells exhibited the most pronounced cell–cell interactions with CD8^+^ T cells, and *in vitro* co−culture experiments confirmed that the immunomodulatory mechanism of UAP1 is functionally associated with CD8^+^ T cell activity. Collectively, these results demonstrate that UAP1 regulates the differentiation and metabolic state of MM cells, thereby promoting immune evasion. These findings provide compelling evidence that UAP1 is a promising therapeutic target, consistent with a recent study (https://papers.ssrn.com/sol3/papers.cfm?abstract_id=5846718).

Although we utilized a substantial sample size and conducted an *in vitro* experimental validation, several limitations persisted. In our study, we demonstrated that UAP1 serves as a significant prognostic and therapeutic biomarker for MM. However, integrating clinical features with established biomarkers remains necessary to fully elucidate UAP1’s impact as a reliable MM biomarker. Additionally, we identified an association between UAP1 expression and LDL levels, raising important questions regarding whether lipid metabolic pathways influence MM pathogenesis (42, 43), which warrant further investigation. UAP1 is elevated in a distinct tumor cell subpopulation, raising the intriguing possibility that these cells represent tumor-initiating or stem-like cells.

Furthermore, three key aspects require clarification: (1) the proportion of patients with high UAP1 expression, and the specific mechanisms by which UAP1 regulates MM cells; Although single-cell analyses suggested that UAP1 may modulate the MIF signaling axis to alter CD8^+^ T cell infiltration and function, MIF transcript levels did not change significantly upon UAP1 knockdown. Given that UAP1 is a key enzyme in the UDP-GlcNAc biosynthesis pathway, and MIF is known to undergo post-translational modifications, it remains formally possible that UAP1 affects MIF function via glycosylation rather than transcriptional regulation, but this hypothesis requires dedicated future investigation. (2) the critical threshold of UAP1 gene levels that affect patient prognosis; and (3) the potential development of UAP1-based personalized treatment approaches. Due to limitations in public database annotations, some specific cytogenetic events (e.g., individual translocations) could not be included in the multivariate model across all cohorts. Future prospective cohorts with complete FISH information are warranted to further validate the independent prognostic value of UAP1 relative to high-risk cytogenetics. Additional *in vitro* and *in vivo* experiments are required to substantiate UAP1’s therapeutic potential.

Nevertheless, our study represents the first large-scale investigation (with > 3,000 samples) to establish the following: (1) the clinical relevance of UAP1 in MM and (2) UAP1’s dual role as both a prognostic indicator and a therapeutic target. We conclusively demonstrated that elevated UAP1 expression correlates with poor prognosis and enhanced treatment response in patients with MM. These findings suggest a novel strategy for the treatment and management of MM. In future clinical settings, UAP1 could be utilized as a prognostic biomarker for predicting patient outcomes and therapeutic efficacy. Moreover, targeting UAP1 represents a promising candidate approach for the treatment of multiple myeloma.

## Data Availability

The original contributions presented in the study are included in the article/**Supplementary Material**, further inquiries can be directed to the corresponding author/s.

## References

[1] Kumar SK Rajkumar SV Dispenzieri A Lacy MQ Hayman SR Buadi FK . Improved survival in multiple myeloma and the impact of novel therapies. Blood. (2008) 111:2516–20. doi: 10.1182/blood-2007-10-116129 17975015 PMC2254544

[2] Landgren O Morgan GJ . Biologic frontiers in multiple myeloma: from biomarker identification to clinical practice. Clin Cancer Res: Off J Am Assoc For Cancer Res. (2014) 20:804–13. doi: 10.1158/1078-0432.ccr-13-2159 24270684 PMC5576179

[3] Levin A Hari P Dhakal B . Novel biomarkers in multiple myeloma. Trans Res J Lab Clin Med. (2018) 201:49–59. doi: 10.1016/j.trsl.2018.05.003 30301522

[4] Chapman MA Lawrence MS Keats JJ Cibulskis K Sougnez C Schinzel AC . Initial genome sequencing and analysis of multiple myeloma. Nature. (2011) 471:467–72. doi: 10.1038/nature09837 21430775 PMC3560292

[5] Pavlova NN Thompson CB . The emerging hallmarks of cancer metabolism. Cell Metab. (2016) 23:27–47. doi: 10.1016/j.cmet.2015.12.006 26771115 PMC4715268

[6] Wang M Zhu J Lubman DM Gao C . Aberrant glycosylation and cancer biomarker discovery: a promising and thorny journey. Clin Chem Lab Med. (2019) 57:407–16. doi: 10.1515/cclm-2018-0379 30138110 PMC6785348

[7] Pinho SS Reis CA . Glycosylation in cancer: mechanisms and clinical implications. Nat Rev Cancer. (2015) 15:540–55. doi: 10.1038/nrc3982 26289314

[8] Josic D Martinovic T Pavelic K . Glycosylation and metastases. Electrophoresis. (2019) 40:140–50. doi: 10.1002/elps.201800238 30246896

[9] Very N Lefebvre T El Yazidi-Belkoura I . Drug resistance related to aberrant glycosylation in colorectal cancer. Oncotarget. (2018) 9:1380–402. doi: 10.18632/oncotarget.22377 29416702 PMC5787446

[10] Fuster MM Esko JD . The sweet and sour of cancer: glycans as novel therapeutic targets. Nat Rev Cancer. (2005) 5:526–42. doi: 10.1038/nrc1649 16069816

[11] Krześlak A Forma E Bernaciak M Romanowicz H Bryś M . Gene expression of O-GlcNAc cycling enzymes in human breast cancers. Clin Exp Med. (2012) 12:61–5. doi: 10.1007/s10238-011-0138-5 PMC3295997 21567137

[12] Lynch TP Ferrer CM Jackson SR Shahriari KS Vosseller K Reginato MJ . Critical role of O-Linked β-N-acetylglucosamine transferase in prostate cancer invasion, angiogenesis, and metastasis. J Biol Chem. (2012) 287:11070–81. doi: 10.1074/jbc.m111.302547 22275356 PMC3322861

[13] Puttamallesh VN Deb B Gondkar K Jain A Nair B Pandey A . Quantitative proteomics of urinary bladder cancer cell lines identify UAP1 as a potential therapeutic target. Genes (Basel). (2020) 11:763. doi: 10.3390/genes11070763 32650368 PMC7397020

[14] Wang X Chen X Liu H . Expression and bioinformatics-based functional analysis of UAP1 in lung adenocarcinoma. Cancer Manag Res. (2020) 12:12111–21. doi: 10.2147/CMAR.S282238 PMC7701148 33269005

[15] Wu XC Yu YZ Zuo YZ Song XL Zhou ZE Xiao Y . Identification of UAP1L1 as a critical factor for prostate cancer and underlying molecular mechanism in tumorigenicity. J Transl Med. (2022) 20:91. doi: 10.1186/s12967-022-03291-0 35168617 PMC8845250

[16] Munkley J . Glycosylation is a global target for androgen control in prostate cancer cells. Endocrine-Related Cancer. (2017) 24:R49–64. doi: 10.1530/erc-16-0569 28159857

[17] Su Z Wang C Pan R Li H Chen J Tan J . The hexosamine biosynthesis pathway-related gene signature correlates with immune infiltration and predicts prognosis of patients with osteosarcoma. Front Immunol. (2022) 13:1028263. doi: 10.3389/fimmu.2022.1028263 36275679 PMC9582954

[18] Itkonen HM Engedal N Babaie E Luhr M Guldvik IJ Minner S . UAP1 is overexpressed in prostate cancer and is protective against inhibitors of N-linked glycosylation. Oncogene. (2015) 34:3744–50. doi: 10.1038/onc.2014.307 25241896

[19] Liu R Gao Q Foltz SM Fowles JS Yao L Wang JT . Co-evolution of tumor and immune cells during progression of multiple myeloma. Nat Commun. (2021) 12:2559. doi: 10.1038/s41467-021-22804-x 33963182 PMC8105337

[20] Hou X Dong Q Hao J Liu M Ning J Tao M . NSUN2-mediated m(5)C modification drives alternative splicing reprogramming and promotes multidrug resistance in anaplastic thyroid cancer through the NSUN2/SRSF6/UAP1 signaling axis. Theranostics. (2025) 15:2757–77. doi: 10.7150/thno.104713 40083919 PMC11898302

[21] Huang J Chan SC Lok V Zhang L Lucero-Prisno DE Xu W . The epidemiological landscape of multiple myeloma: a global cancer registry estimate of disease burden, risk factors, and temporal trends. Lancet Haematol. (2022) 9:e670–7. doi: 10.1016/s2352-3026(22)00165-x 35843248

[22] Bao A Zhao Q Merritt E Bumma N Devarakonda S Khan AM . Racial differences as predictors of outcomes in young patients with multiple myeloma. Blood Cancer J. (2022) 12:114. doi: 10.1038/s41408-022-00708-3 35896527 PMC9329432

[23] Dragoș ML Ivanov IC Mențel M Văcărean-Trandafir IC Sireteanu A Titianu AA . Prognostic value of association of copy number alterations and cell-surface expression markers in newly diagnosed multiple myeloma patients. Int J Mol Sci. (2022) 23:7350. doi: 10.3390/ijms23147530 35886877 PMC9318311

[24] Brigle K Rogers B . Pathobiology and diagnosis of multiple myeloma. Semin Oncol Nurs. (2017) 33:225–36. doi: 10.1016/j.soncn.2017.05.012 28688533

[25] Chi KR . The tumour trail left in blood. Nature. (2016) 532:269–71. doi: 10.1038/532269a 27075102

[26] Zhu B Ju S Chu H Shen X Zhang Y Luo X . The potential function of microRNAs as biomarkers and therapeutic targets in multiple myeloma. Oncol Lett. (2018) 15:6094–106. doi: 10.3892/ol.2018.8157 29731841 PMC5920744

[27] Valcárcel LV San José-Enériz E Ordoñez R Apaolaza I Olaverri-Mendizabal D Barrena N . An automated network-based tool to search for metabolic vulnerabilities in cancer. Nat Commun. (2024) 15:8685. doi: 10.1038/s41467-024-52725-4 39394196 PMC11470099

[28] Cagnetta A Lovera D Grasso R Colombo N Canepa L Ballerini F . Mechanisms and clinical applications of genome instability in multiple myeloma. BioMed Res Int. (2015) 2015:943096. doi: 10.1155/2015/943096 26579543 PMC4633548

[29] Biran N Jagannath S Chari A . Risk stratification in multiple myeloma, part 1: characterization of high-risk disease. Clin Adv Hematol Oncol H&O. (2013) 11:489–503. 24518420

[30] Sawyer JR Tian E Walker BA Wardell C Lukacs JL Sammartino G . An acquired high-risk chromosome instability phenotype in multiple myeloma: Jumping 1q syndrome. Blood Cancer J. (2019) 9:62. doi: 10.1038/s41408-019-0226-4 31399558 PMC6689064

[31] Bisht K Walker B Kumar SK Spicka I Moreau P Martin T . Chromosomal 1q21 abnormalities in multiple myeloma: a review of translational, clinical research, and therapeutic strategies. Expert Rev Hematol. (2021) 14:1099–114. doi: 10.1080/17474086.2021.1983427 34551651

[32] Zhan F Huang Y Colla S Stewart JP Hanamura I Gupta S . The molecular classification of multiple myeloma. Blood. (2006) 108:2020–8. doi: 10.1182/blood-2005-11-013458 16728703 PMC1895543

[33] Li C Wendlandt EB Darbro B Xu H Thomas GS Tricot G . Genetic analysis of multiple myeloma identifies cytogenetic alterations implicated in disease complexity and progression. Cancers. (2021) 13:517. doi: 10.3390/cancers13030517 33572851 PMC7866300

[34] Palumbo A Avet-Loiseau H Oliva S Lokhorst HM Goldschmidt H Rosinol L . Revised international staging system for multiple myeloma: a report from international myeloma working group. J Clin Oncol Off J Am Soc Clin Oncol. (2015) 33:2863–9. doi: 10.1200/jco.2015.61.2267 26240224 PMC4846284

[35] Alagpulinsa DA Kumar S Talluri S Nanjappa P Buon L Chakraborty C . Amplification and overexpression of E2 ubiquitin conjugase UBE2T promotes homologous recombination in multiple myeloma. Blood Adv. (2019) 3:3968–72. doi: 10.1182/bloodadvances.2019000181 31805191 PMC6963244

[36] Cheng Y Sun F Alapat DV Wanchai V Mery D Siegel ER . Multi-omics reveal immune microenvironment alterations in multiple myeloma and its precursor stages. Blood Cancer J. (2024) 14:194. doi: 10.1038/s41408-024-01172-x 39505839 PMC11541562

[37] Zavidij O Haradhvala NJ Mouhieddine TH Sklavenitis-Pistofidis R Cai S Reidy M . Single-cell RNA sequencing reveals compromised immune microenvironment in precursor stages of multiple myeloma. Nat Cancer. (2020) 1:493–506. doi: 10.1182/blood-2018-99-114145 33409501 PMC7785110

[38] He D Wang D Lu P Yang N Xue Z Zhu X . Single-cell RNA sequencing reveals heterogeneous tumor and immune cell populations in early-stage lung adenocarcinomas harboring EGFR mutations. Oncogene. (2021) 40:355–68. doi: 10.1038/s41388-020-01528-0 33144684 PMC7808940

[39] Kuang J Zhong G Zhao L Yuan X Zhou Y Li J . Machine learning analysis reveals tumor heterogeneity and stromal-immune niches in breast cancer. NPJ Digit Med. (2025) 8:565. doi: 10.1038/s41746-025-01967-7 40897851 PMC12405446

[40] Onieva JL Xiao Q Berciano-Guerrero MÁ Laborda-Illanes A de Andrea C Chaves P . High IGKC-expressing intratumoral plasma cells predict response to immune checkpoint blockade. Int J Mol Sci. (2022) 23:9124. doi: 10.3390/ijms23169124 36012390 PMC9408876

[41] Zheng C Wang J Zhou Y Duan Y Zheng R Xie Y . IFNα-induced BST2(+) tumor-associated macrophages facilitate immunosuppression and tumor growth in pancreatic cancer by ERK-CXCL7 signaling. Cell Rep. (2024) 43:114088. doi: 10.1016/j.celrep.2024.114088 38602878

[42] Dong H Chen J Zhang H Zhao M Yue Y Wang S . Palmitic acid inhibits macrophage-mediated chemotherapy resistance in multiple myeloma via ALOX12 signaling. Int Immunopharmacol. (2024) 143:113320. doi: 10.1016/j.intimp.2024.113320 39378653

[43] Yan W Shi X Zhao Y Liu X Jia X Gao L . Microbiota-reprogrammed phosphatidylcholine inactivates cytotoxic CD8 T cells through UFMylation via exosomal SerpinB9 in multiple myeloma. Nat Commun. (2025) 16:2863. doi: 10.1038/s41467-025-57966-5 40128181 PMC11933704

