# Supplementary material for: UAP1 as a prognostic biomarker regulating malignant biological functions in multiple myeloma cells
Source: Front Oncol. 2026 Jul 22;16:1902414. doi: 10.3389/fonc.2026.1902414 (PMC13437300; doi:10.3389/fonc.2026.1902414)
Supplement: Supplementary file 1 [file SupplementaryFile1.pdf]

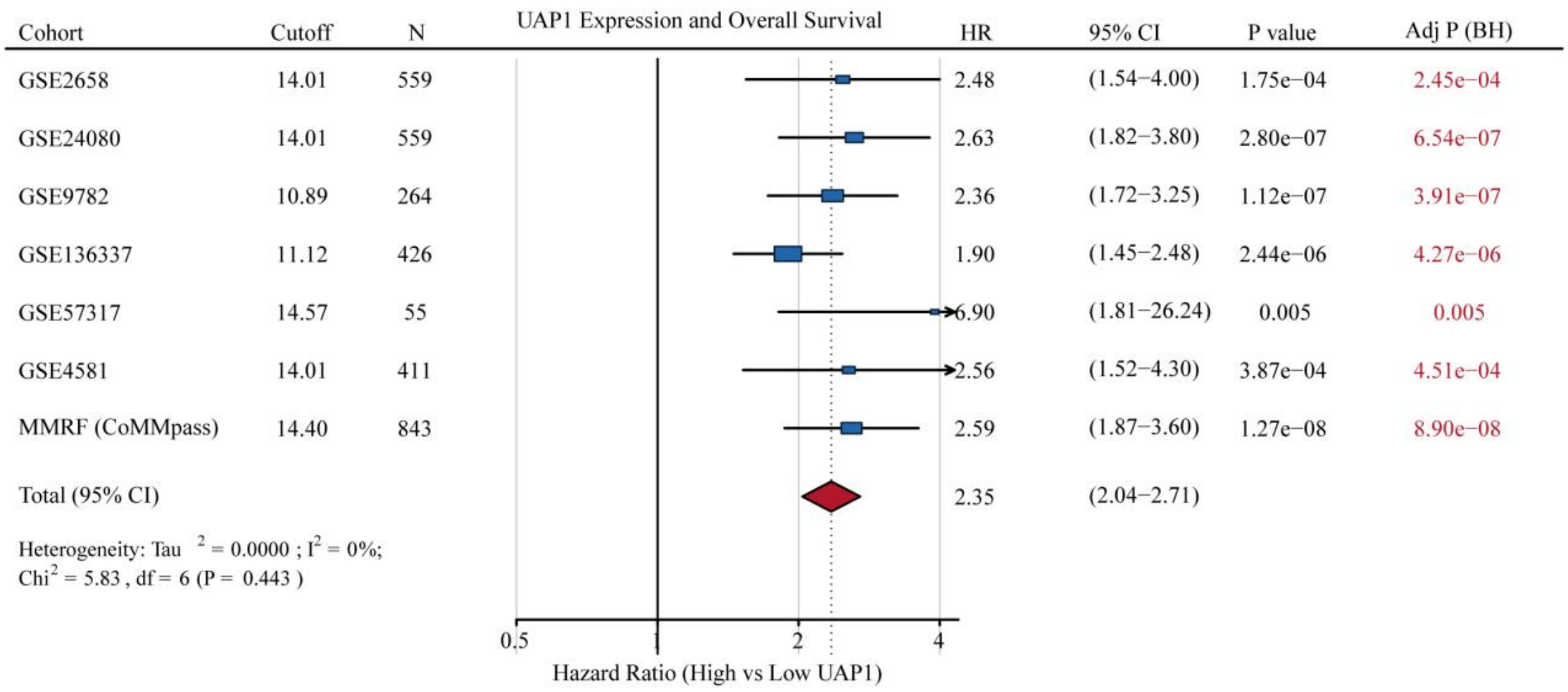

Supplementary Figure 1. Meta-analysis of UAP1 as a Risk Factor for Multiple Myeloma

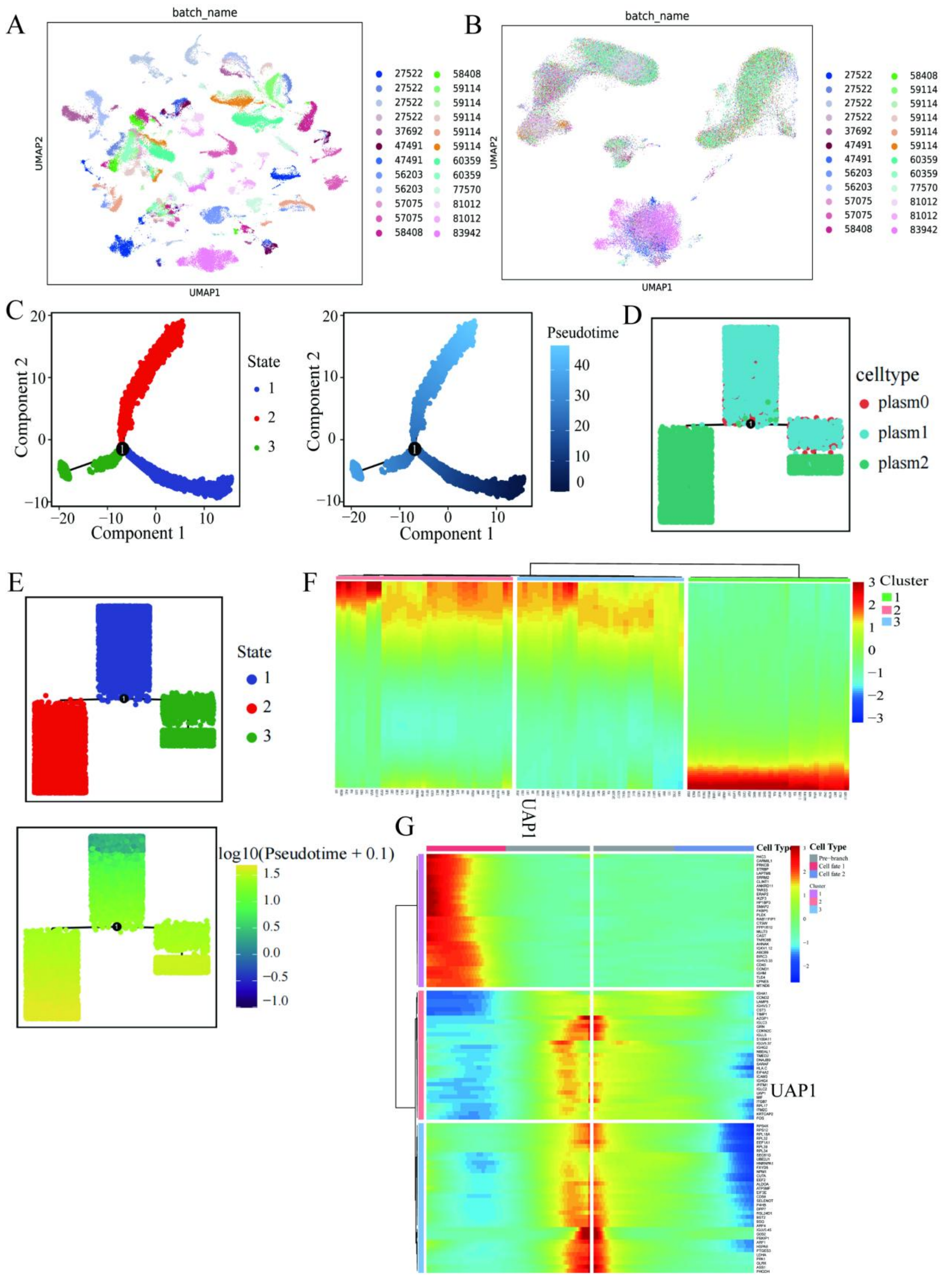

Supplementary Figure 2. Trajectory analysis and clustering heat map

A: UMAP dimensionality reduction analysis (unadjusted for batch effects); **B:** Batch effect-corrected UMAP visualization (color-coded by sample); **C:** Pseudotime trajectory analysis (cell states vs. developmental progression); **D-E:** Trajectory-state correlation analysis across cell types; F: Trajectory analysis and gene expression clustering heatmap; G: Heatmap of gene expression clustering before and after the trajectory analysis inflection point.

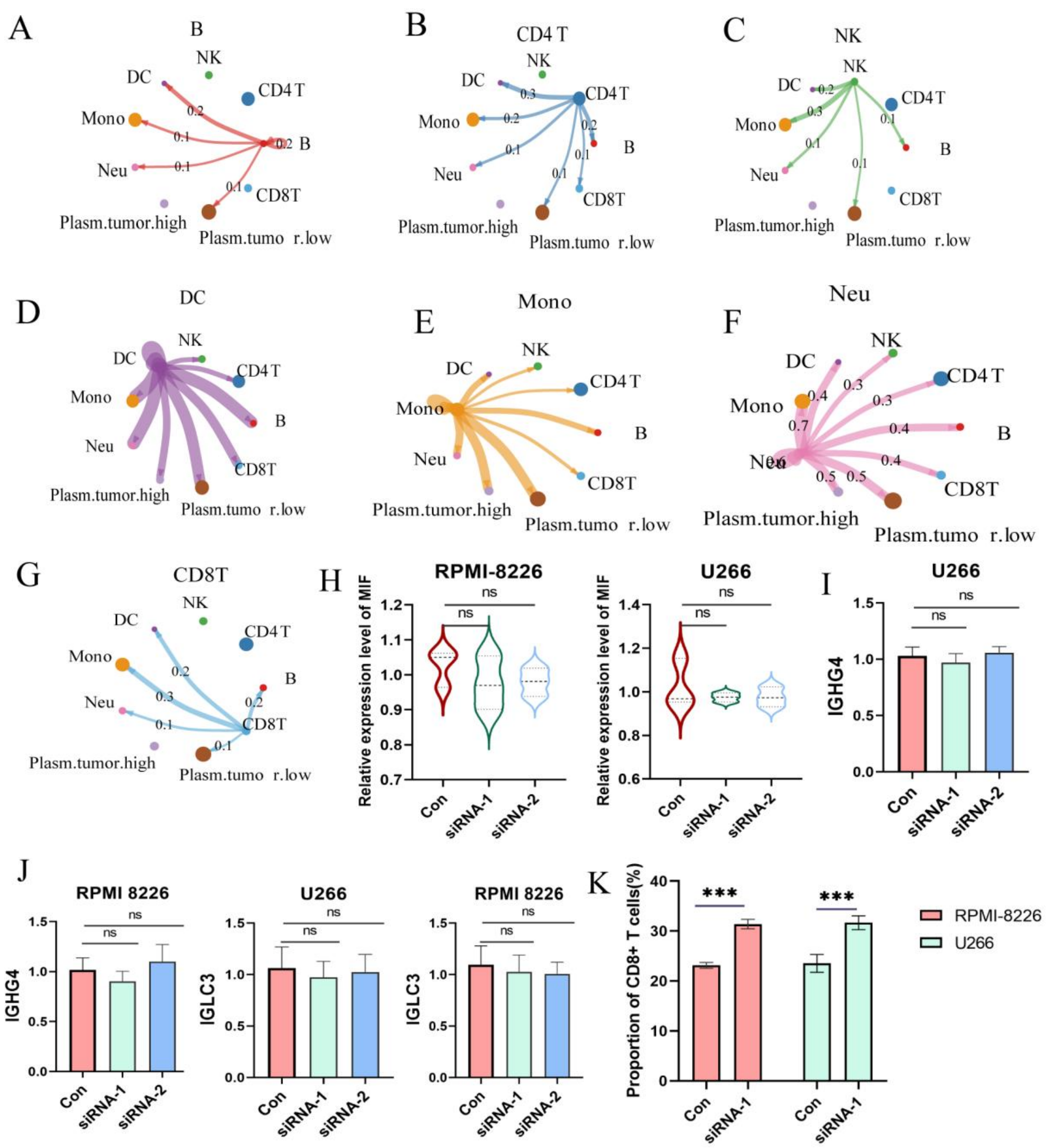

Supplementary Figure 3

A-G: Correlation of cellular communication with tumour status. This panel shows the interaction networks between immune cell subtypes and their corresponding tumour states (Plasm.tumor.high vs. Plasm.tumor.low). (D) Cell populations analyzed include DC (Dendritic Cell), (A) B (B Cell), (B) CD4T (CD4+ T Cell), (C) NK (Natural Killer Cell), (E) Mono (Monocyte), (F) Neu (Neutrophil), (G) CD8T (Cytotoxic T Cell); H-J: Expression levels of MIF, IGHG4 and IGLC3 genes after UAP1 gene knockdown; K: Statistical analysis of CD8+T ratios in co-culture experiments. ($*P<0.05$, $**P<0.01$, $***P<0.001$; All experiments were repeated 3 times).
