# Supplementary material for: UAP1 as a prognostic biomarker regulating malignant biological functions in multiple myeloma cells
Source: Front Oncol. 2026 Jul 22;16:1902414. doi: 10.3389/fonc.2026.1902414 (PMC13437300; doi:10.3389/fonc.2026.1902414)
Supplement: Supplementary file 2 [file SupplementaryFile2.pdf]

Fig 4D

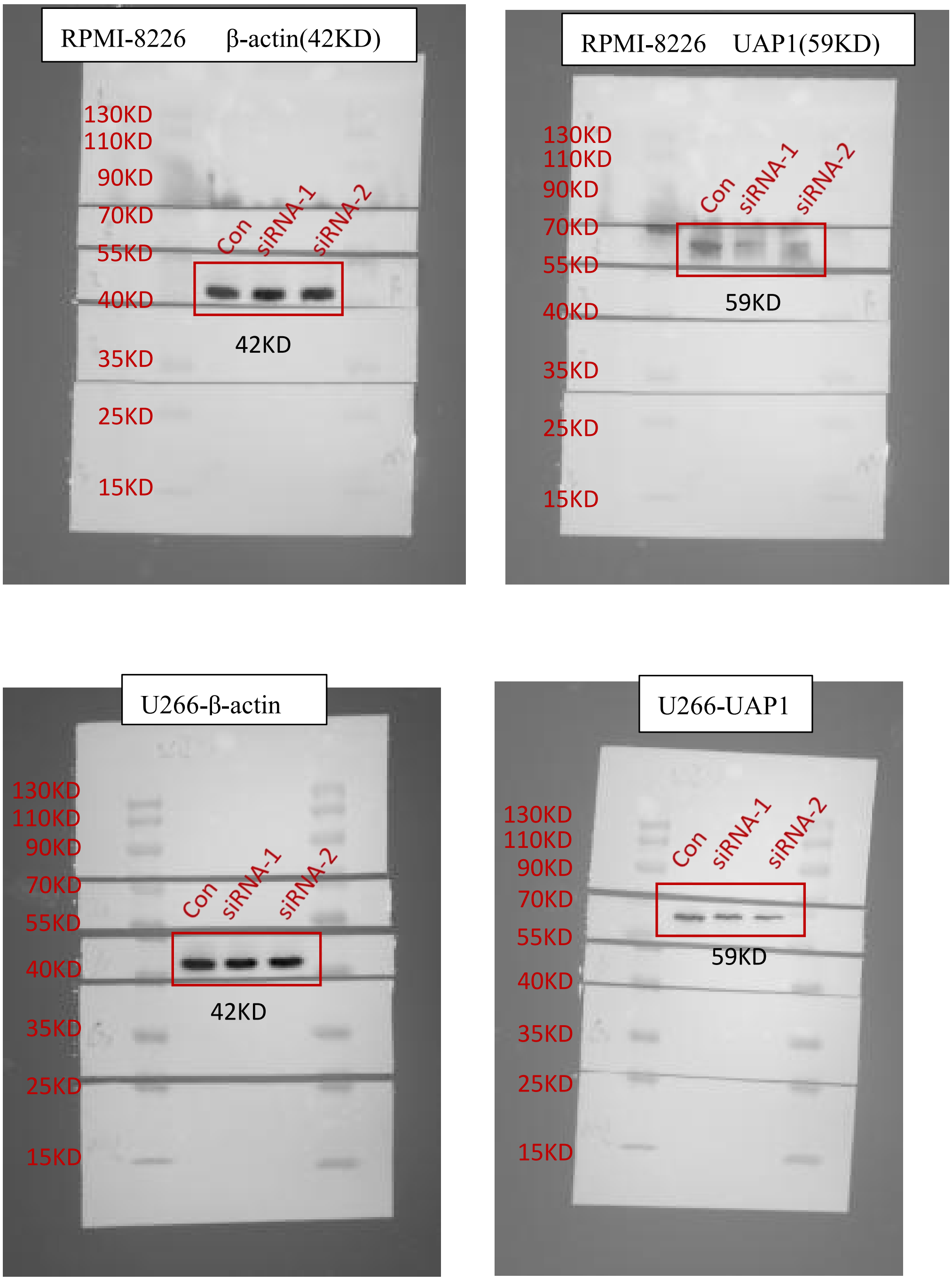
RPMI-8226 β-actin(42KD)
130KD
110KD
90KD
70KD
55KD
40KD
35KD
25KD
15KD
Con
siRNA-1
siRNA-2
42KD
RPMI-8226 UAP1(59KD)
130KD
110KD
90KD
70KD
55KD
40KD
35KD
25KD
15KD
Con
siRNA-1
siRNA-2
59KD
U266-β-actin
130KD
110KD
90KD
70KD
55KD
40KD
35KD
25KD
15KD
Con
siRNA-1
siRNA-2
42KD
U266-UAP1
130KD
110KD
90KD
70KD
55KD
40KD
35KD
25KD
15KD
Con
siRNA-1
siRNA-2
59KD

Fig 4I

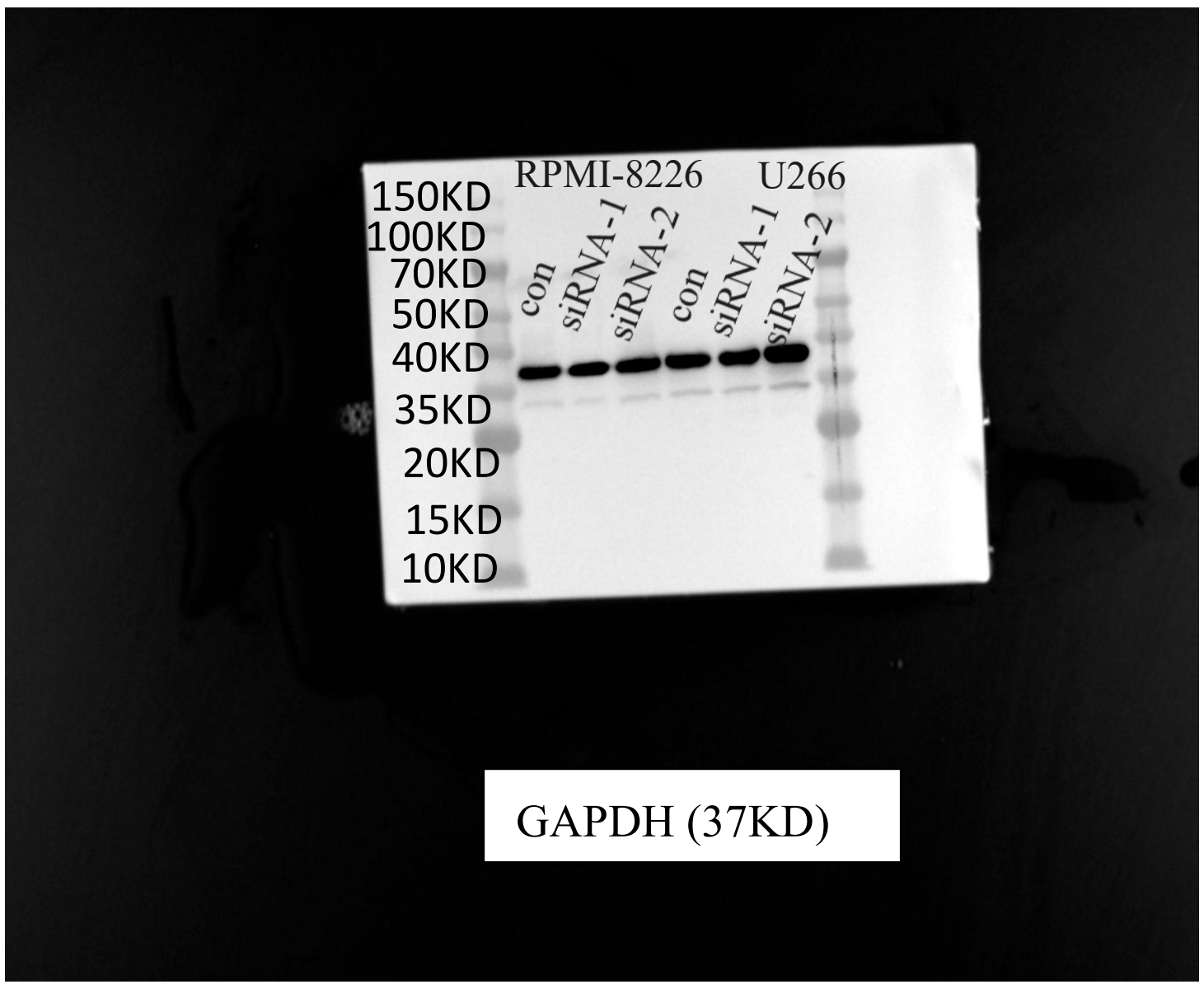
150KD
100KD
70KD
50KD
40KD
35KD
20KD
15KD
10KD
RPMI-8226
U266
con
siRNA-1
siRNA-2
con
siRNA-1
siRNA-2
GAPDH (37KD)

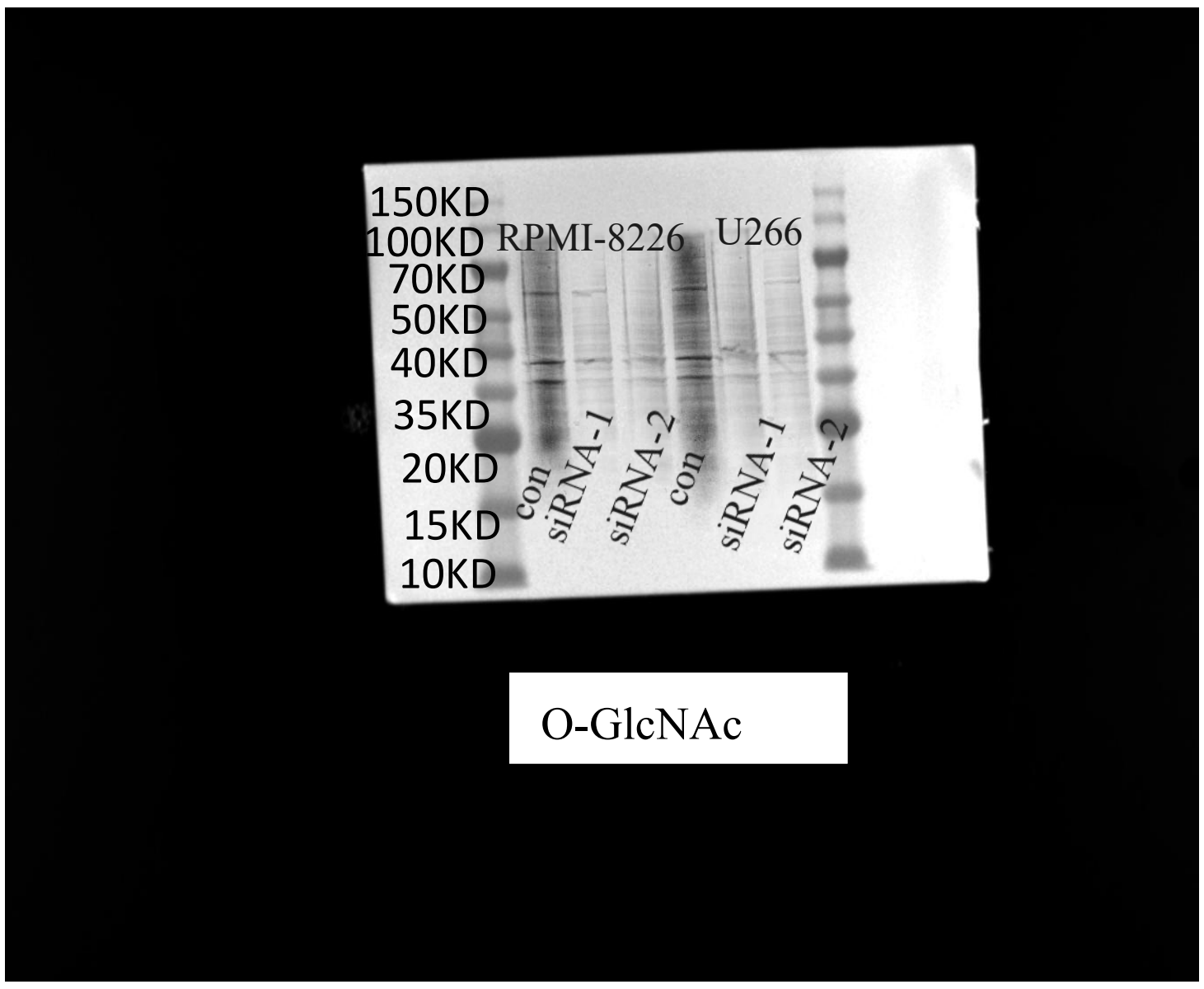
150KD
100KD
70KD
50KD
40KD
35KD
20KD
15KD
10KD
RPMI-8226
U266
con
siRNA-1
siRNA-2
con
siRNA-1
siRNA-2
O-GlcNAc
