# Supplementary material for: UAP1 as a prognostic biomarker regulating malignant biological functions in multiple myeloma cells
Source: Front Oncol. 2026 Jul 22;16:1902414. doi: 10.3389/fonc.2026.1902414 (PMC13437300; doi:10.3389/fonc.2026.1902414)
Supplement: Supplementary file 3 [file SupplementaryFile4.docx]

**2.5 Single-cell Data Collection and Processing**

We obtained single-cell samples from bone marrow aspirates of 24 multiple myeloma patients (studying tumor-immune cell co-evolution during disease progression), yielding a total of 74,986 single cells. As mentioned in our introduction, these single-cell data were derived from the study by Liu et al. and are publicly available. Quality control was performed by: (1) excluding cells with >15% mitochondrial gene content, (2) retaining only cells with >1,000 total reads and >500 detected genes, and (3) removing doublets identified using the Scrublet algorithm (default parameters). Batch effects were corrected using the Harmony algorithm prior to downstream analysis. Cell clusters were identified through standard dimensionality reduction and clustering approaches, with cell type annotation performed using established marker genes. All analyses were conducted using the Scanpy software package.

**2.5.1 Differential Expression Analysis**

To identify differentially expressed genes (DEGs) across distinct cell types or patient subgroups, we performed differential expression analysis using the Presto R package. Results were visualized using ggplot2 (R). In addition, in the datasets we analyzed, patients were divided into high- and low-UAP1 expression groups based on the median UAP1 expression level. To determine the optimal cutoff value for prognostic analysis, we also used the surv_cutpoint function from the survminer R package, which identifies the optimal cutoff by maximizing the log-rank statistic for survival differences.

**2.5.2 Gene Set Enrichment Analysis (GSEA)**

Functional enrichment analysis was conducted using the clusterProfiler package, with gene ontology biological processes (GOBP) from the MSigDB (Molecular Signatures Database). Enrichment results were then visualized for interpretation.

**2.5.3 Pseudotime Trajectory Analysis Using Monocle2**

To investigate the association between UAP1 expression and the progression of distinct tumor subtypes, we performed single-cell trajectory analysis using Monocle2 (R package). Cell developmental potential was first estimated with CytoTRACE2, and the subpopulation exhibiting the highest developmental potential was designated as the trajectory’ s root state. The analysis included: Data preprocessing (quality filtering, normalization); Trajectory inference (dimensionality reduction, minimum spanning tree construction); Visualization (UMAP/t-SNE embedding); Dynamic gene expression profiling along pseudotime.

**2.5.4 Cell-Cell Communication Analysis with CellChat**

To systematically characterize interactions between UAP1-high multiple myeloma (MM) tumor cells (from treatment-naive patients) and other cell types, we employed CellChat for ligand-receptor network analysis. The pipeline included: Data object creation (createCellChat() function); Communication probability estimation (computeCommunProb()); Pathway-level interaction analysis (computeCommunProbPathway()); Visualization of dominant signaling pathways.
